# Cell2Grid: an efficient, spatial, and convolutional neural network-ready representation of cell segmentation data

**DOI:** 10.1117/1.JMI.9.6.067501

**Published:** 2022-11-30

**Authors:** Laurin Herbsthofer, Martina Tomberger, Maria A. Smolle, Barbara Prietl, Thomas R. Pieber, Pablo López-García

**Affiliations:** a CBmed, Center for Biomarker Research in Medicine GmbH, Graz, Austria; b BioTechMed, Graz, Austria; c Medical University of Graz, Department of Orthopaedics and Trauma, Graz, Austria; d Medical University of Graz, Division of Endocrinology and Diabetology, Graz, Austria; e Health Institute for Biomedicine and Health Sciences, Joanneum Research Forschungsgesellschaft mbH, Graz, Austria

**Keywords:** medical imaging, image processing, neural networks, image segmentation, image compression, image storage

## Abstract

**Purpose:**

Cell segmentation algorithms are commonly used to analyze large histologic images as they facilitate interpretation, but on the other hand they complicate hypothesis-free spatial analysis. Therefore, many applications train convolutional neural networks (CNNs) on high-resolution images that resolve individual cells instead, but their practical application is severely limited by computational resources. In this work, we propose and investigate an alternative spatial data representation based on cell segmentation data for direct training of CNNs.

**Approach:**

We introduce and analyze the properties of Cell2Grid, an algorithm that generates compact images from cell segmentation data by placing individual cells into a low-resolution grid and resolves possible cell conflicts. For evaluation, we present a case study on colorectal cancer relapse prediction using fluorescent multiplex immunohistochemistry images.

**Results:**

We could generate Cell2Grid images at 5-μm resolution that were 100 times smaller than the original ones. Cell features, such as phenotype counts and nearest-neighbor cell distances, remain similar to those of original cell segmentation tables (p<0.0001). These images could be directly fed to a CNN for predicting colon cancer relapse. Our experiments showed that test set error rate was reduced by 25% compared with CNNs trained on images rescaled to 5μm with bilinear interpolation. Compared with images at 1-μm resolution (bilinear rescaling), our method reduced CNN training time by 85%.

**Conclusions:**

Cell2Grid is an efficient spatial data representation algorithm that enables the use of conventional CNNs on cell segmentation data. Its cell-based representation additionally opens a door for simplified model interpretation and synthetic image generation.

## 1 Introduction

Convolutional neural networks (CNNs) have revolutionized image analysis and are considered the *de facto* standard for image classification tasks.^1^^–^^5^ Their main strength is the ability to learn complex spatial patterns directly from labeled examples without the need for prior knowledge or manual feature extraction. The practical application of CNNs, however, depends on several factors, the size of input images being one of the most limiting. As an example, ImageNet, which is a widespread academic resource for annotated image data,^6^ contains images that are less than a megapixel in size on average and are even further cropped and resized to 256×256 before training CNNs. In contrast, medical images, especially whole-slide imaging (WSI) of tissue sections, can be many orders of magnitude larger, ranging from multimegapixel image tiles or regions-of-interest (ROI) to gigapixels for WSI,^7^ making their direct analysis using CNNs unfeasible due to computational constraints.

Even though conventional image rescaling is frequently used in other imaging domains,^1^ it is rarely applied to histologic images because small details of individual cells might be lost, eliminating the advantages of microscope magnification. Therefore, most biomedical applications using CNNs on histologic images derived from hematoxylin and eosin (H&E)^8^^–^^10^ and fluorescent multiplex immunohistochemistry (fm-IHC)^11^^–^^17^ have relied on tiling or patching^18^^–^^20^ as the preferred image preprocessing step. However, extensive image tiling leads to new challenges, including loss of large-scale image context, weakly supervised labels, and multiinstance learning settings. Researchers are actively proposing new solutions to these problems,^21^^–^^23^ but they are still not general enough to be applied systematically.

An alternative and more classical approach to using histologic images is to focus on individual cells instead.^15^^,^^16^^,^^24^^–^^27^ Cell segmentation algorithms take a histologic image as input and generate a list of identified cells with their coordinates in the image and other features (e.g., cell size, shape, texture, antibody marker expression levels and others). Many cell segmentation algorithms are already implemented in open source and commercial software (e.g., CellProfiler,^28^ ImageJ,^29^ and inForm (Akoya Biosciences, Inc.), or HALO software (Indica Labs)) and new ones are published every year.^30^^,^^31^ Cell segmentation allows one to perform more traditional hypothesis-driven spatial analysis, including cell phenotype quantification in different tissue regions, nearest neighbor analysis, identification of touching cells and cell clusters, and phenotype colocalization.^32^^–^^35^ In fact, several biomarkers have been identified this way, indicating the importance of cell segmentation in tissue imaging.^36^^–^^39^ However, in contrast to feeding histologic images to CNNs directly, this kind of analysis requires a substantial amount of a priori domain knowledge to extract meaningful spatial features.

Most importantly, some biological questions still remain elusive to such hypothesis-driven approaches, as is the case of colorectal cancer relapse and survival prediction.^40^^–^^44^ In such cases, machine learning methods, such as random forests, support vector machines, or neural networks could be helpful, but these algorithms cannot directly consume the output of cell segmentation algorithms (one table per sample). To overcome this limitation, graph neural networks (GNNs),^45^^,^^46^ which first convert the list of identified cells into a so-called cell graph that represents cell–cell interactions in the data,^47^ have been proposed. Recent research explored the design space for GNNs for graph classification, highlighting the complexity of choosing an appropriate GNN for any given task.^48^ Additionally, several hyperparameters for the construction of cell graphs (usually the parameters for k-nearest neighbor connections of cells and a maximum edge length cutoff^46^) require careful tuning. The identification of best-practices for constructing cell graphs and the selection of a suitable GNN architecture therefore remain active fields of research.^48^^–^^50^ Compared with these graph-based methods, conventional pixel-based CNNs are a mature technology that is well established in biomedical tissue analysis.^51^^,^^52^

In this paper, we introduce Cell2Grid, an algorithm for efficient representation of histologic images that transform cell segmentation data into low-resolution images suitable for training conventional CNNs. To the best of our knowledge, this is a novel approach that has not been explored in depth. For evaluation, we present a case study on colorectal cancer relapse prediction where we show that Cell2Grid images maintain cell spatial information while providing better performance compared with conventional image rescaling when training CNNs.

## 2 Methods

**Fig. 1:**
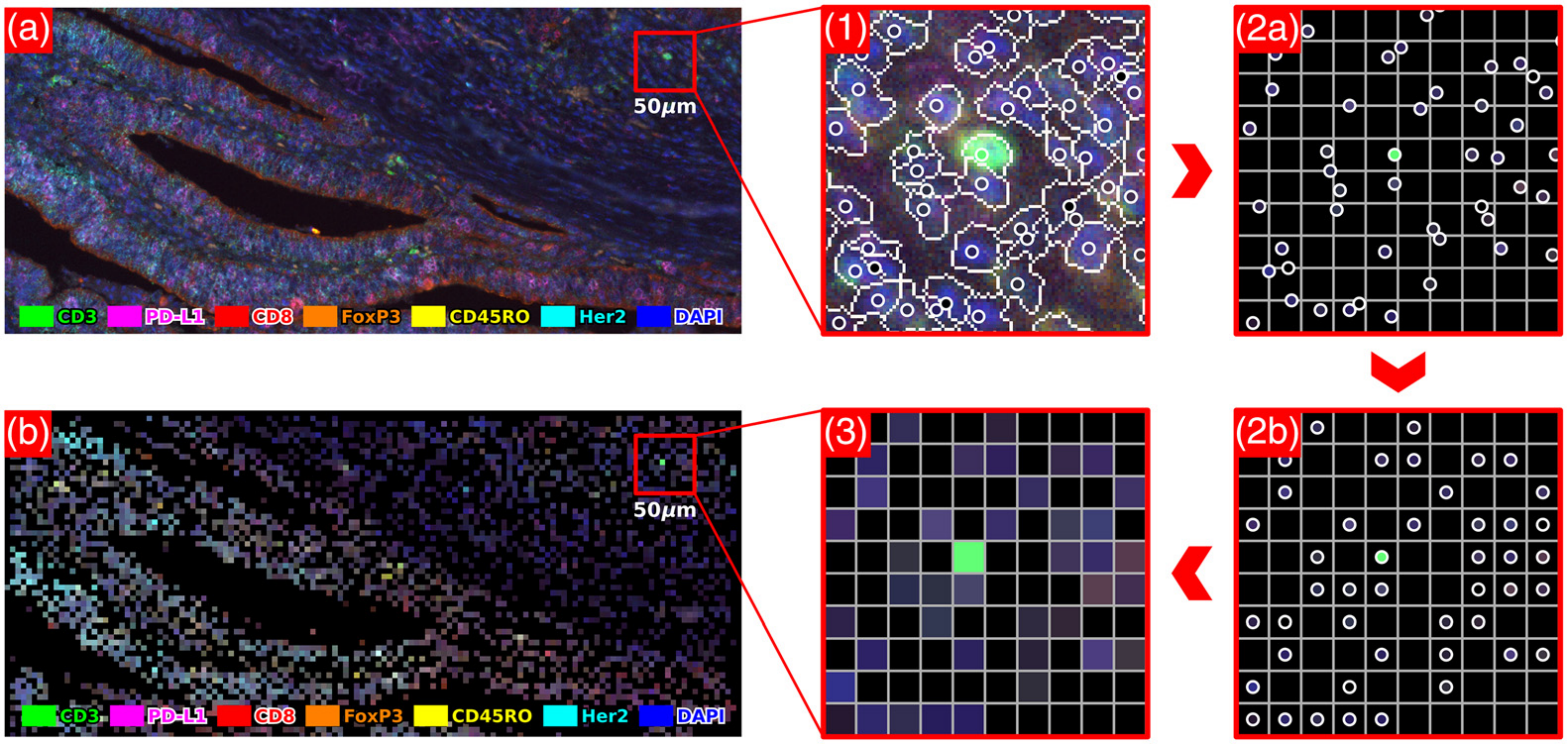
Visualization of the image-to-image pipeline of Cell2Grid. (a) Input fm-IHC image (0.5  μm/px) with six fluorescence color channels + DAPI, shown as false-color RGB image; (1) cell segmentation with cell nuclei as circles; (2a) location of cells relative to 5  μm target grid; (2b) one-to-one assignment of cells to target grid; (3) Cell2Grid data representation; and (b) final Cell2Grid image (5  μm/px, 100 times smaller compared with original data size), shown as false-color RGB image (best viewed on screen).

Cell2Grid transforms the table-style output of cell segmentation methods into spatial cell-based data representations (referred to as “Cell2Grid images”) that are orders of magnitudes smaller than original input images. The entire image-to-image pipeline consists of three steps (see Fig. 1): (1) cell segmentation and cell feature extraction, (2) assignment of cells to a target grid, and (3) image creation. Step 2 is the main contribution of this work and depends on two parameters, such as the target grid spacing d and the maximum local conflict resolution size $w_{\max}$, which controls how cell assignment conflicts are resolved. Even though our method is more closely related to image rescaling rather than image compression, we will use the term compression ratio for the ratio between high-resolution input image and Cell2Grid output image sizes as a result of a change in pixel resolution.

### 2.1 Step 1: Cell Segmentation and Feature Extraction

Individual biological cells are identified in the input image using a cell segmentation method suited for the tissue type, see e.g., Refs. 24, 53, and 54. For each identified cell, the location $\vec{X}=(x_{1},x_{2})$ of its nucleus in the image is stored together with f extracted cell features, e.g., the average marker intensity for each fluorescent color channel, the cells size and shape, etc. The final output of the cell segmentation step is a list of cells with their coordinates $\vec{X}$ and f features, as shown in Table 1.

**Table 1:** Example output of the cell identification (Step 1).

Cell ID	$x_{1}$ (μm)	$x_{2}$ (μm)	Feature 1	…	Feature f
1	42.0	3.5	233		93
2	65.5	12.5	20		209
…	…	…	…		…

Cell segmentation in an fm-IHC laboratory setting is normally performed using established software, e.g., CellProfiler,^28^ ImageJ,^29^ inForm (Akoya Biosciences, Inc.), or HALO (Indica Labs), with supervision from an expert in the field, as the process depends on multiple parameters, such as tissue type and tissue stains, and could require manual annotation as training data. This was the setting and scope of our study, but it is worth mentioning that recently published state-of-the-art methods on cell segmentation^30^^,^^31^ might soon become widespread in such a setting, providing even better results (see Sec. 4.1).

### 2.2 Step 2: Assignment of Cells to Target Grid

After choosing a target grid spacing d, all identified cells are assigned to the square target grid by binning their original coordinates $\vec{X}$ to the nodes of the grid using ${\overset{\to }{X}}_{g}=\lfloor \overset{\to }{X}/d\rceil$, whereas ⌊·⌉ denotes conventional rounding to the next integer and ${\vec{X}}_{g}$ corresponds to coordinates of the target grid nodes (GNs) (i.e., indices of pixel locations in the final output image). While for most biological tissue types, d=5  μm is a good choice, we provide guidelines for how to systematically chose the target grid spacing d in Supplementary Material S.3.

Using this method, any two cells within a distance of d√2 may be assigned to the same GN ${\vec{X}}_{g}$. To achieve a one-to-one relation of cells to output pixels, we require that each cell be assigned uniquely to a GN and that any GN can hold at most one biological cell. This one-to-one relation is the essential property of Cell2Grid data representation.

Assignment conflicts are resolved successively by applying Munkres’ algorithm^55^ to all cells within the local 3×3 subgrid around a GN with conflicts. If the number of cells k within this window is higher than the number of GNs in the subgrid, the 5×5 subgrid is considered. This process is continued until a maximum window size $w_{\max}\times w_{\max}$ is reached. If the number of cells still exceeds the number of available GNs, $k>{\left(w_{\max}\right)}^{2}$, then $k-(w_{\max}{)}^{2}$ cells are deleted from the data. This process is explained in more detail in Supplementary Material S.1.

After conflict resolution, every cell has either been uniquely assigned to a GN or was deleted due to unresolvable conflicts. Similarly, every target GN either holds exactly one or no cells.

#### 2.2.1 Grid spacing and assignment conflicts – theoretical analysis

In this section, we present analytical expressions for the expected number of GNs with assignment conflicts and the total expected cell loss after local conflict resolution. These expressions assume a random uniform distribution of cell locations over the entire image and depend on the overall cell density ρ, the target grid spacing d, and the local conflict resolution window size w.

Using results from the ball-into-bin model that describes the occupancy problem,^56^ the random allocation of n cells into m nodes is described by the Poisson distribution $\mathrm{Pois}(\lambda ,X=k)=\frac{\lambda^{k}}{k!}e^{-\lambda }$, which is the probability mass function for a single bin (GN) containing k balls (cells) with $\lambda =\frac{n_{\text{cells}}}{m_{\text{nodes}}}$, and $m_{\text{nodes}}$ being the number of GNs of the target grid. Therefore, Pois(λ,X≥2) estimates the fraction of GNs that hold two or more cells (i.e., assignment conflicts) and is only dependent on λ. For our purpose, λ can be defined using the cell density $\rho =\frac{n_{\text{cells}}}{A}$ of $n_{\text{cells}}$ in an image area A and target grid spacing d as $\lambda (d)=\frac{n_{\text{cells}}}{m_{\text{nodes}}}=\frac{n_{\text{cells}}}{\left(A/d^{2}\right)}=\rho d^{2}$. Therefore, the estimated fraction of GNs with k assigned cells is written as

$$\mathrm{Pois}(\lambda (d),k)=\frac{{\left(\rho d^{2}\right)}^{k}}{k!}e^{-\rho d^{2}}.$$ (1)

Using this result, we express the fraction of GNs that hold two or more cells (i.e., nodes with assignment conflicts) as follows:

$${\mathrm{GN}}_{\mathrm{conf}}(d)=\mathrm{Pois}(\lambda (d),k\geq 2)=1-\mathrm{Pois}(\lambda (d),k=0)-\mathrm{Pois}(\lambda (d),k=1).$$ (2)

The fraction of cells in conflict is the number of cells that are not allocated to one-cell GNs,

$$C_{\mathrm{conf}}(d)=1-\frac{m_{\text{nodes}}}{n_{\text{cells}}}\;\mathrm{Pois}(\lambda (d),k=1).$$ (3)

Next, we estimate the number of unresolvable conflicts when a local conflict resolution window of size w is used (w being an odd integer ≥3). Using a modified target grid spacing $d_{w}=w\cdot d$, we can estimate the number of cells in a w×w subgrid around a GN with conflicts. Subgrids (or “neighborhoods”) that contain more than $w^{2}$ cells constitute unresolvable conflicts. Because taking the local neighborhood into account is only relevant when the central node actually contains a conflict, we multiply with ${\mathrm{GN}}_{\mathrm{conf}}(d)$. By neglecting conditional probabilities for $k>w^{2}$ when there are already k≥2 cells at the central node, we arrive at

$${\mathrm{GN}}_{\mathrm{unres}}(d,w)\approx {\mathrm{GN}}_{\mathrm{conf}}(d,w)\cdot \mathrm{Pois}(\lambda (wd),k>w^{2})\;={\mathrm{GN}}_{\mathrm{conf}}(d,w)\cdot (1-\overset{k=w^{2}}{\underset{k=0}{\sum }}\mathrm{Pois}(\lambda (wd),k)).$$ (4)

Finally, we estimate the cell-loss-fraction due to unresolvable conflicts by considering cells that have been assigned successfully using $C_{\text{loss}}=(n_{\text{cells}}-N_{1}-N_{2})/n_{\text{cells}}$. Here, $N_{1}$ is the number of cells located in “unsaturated” local neighborhoods (all cells are assigned if an area of size $(wd{)}^{2}$ contains $k\leq w^{2}$ cells):

$$N_{1}=m_{\text{nodes}}\overset{w^{2}}{\underset{k=0}{\sum }}\frac{k}{w^{2}}\;\mathrm{Pois}(\lambda (wd),k).$$ (5)

Note that the summation term contains $k/w^{2}$, which is the average number of cells per GN when k cells are in an area of $w^{2}$ GNs. In contrast, $N_{2}$ is the number of cells that are successfully assigned during conflict resolution in “saturated” areas (in areas of size ${\left(wd\right)}^{2}$ containing $k>w^{2}$ cells only $w^{2}$ cells are assigned and $k-w^{2}$ cells need to be deleted):

$$N_{2}=m_{\text{nodes}}\cdot \mathrm{Pois}(\lambda (wd),k>w^{2})=m_{\text{nodes}}(1-\overset{w^{2}}{\underset{k=0}{\sum }}\mathrm{Pois}(\lambda (wd),k)).$$ (6)

In $N_{2}$, we simply count the number of GNs that are part of oversaturated areas because each of them will contain exactly one cell after conflict resolution. Put together and simplified, the estimated cell loss after conflict resolution is

$$C_{\text{loss}}(w,d)=1-\frac{w^{2}}{\lambda (wd)}-\frac{w^{2}}{\lambda (wd)}\overset{w^{2}}{\underset{k=0}{\sum }}\mathrm{Pois}(\lambda (wd),k)\cdot (\frac{k}{w^{2}}-1).$$ (7)

**Fig. 2:**
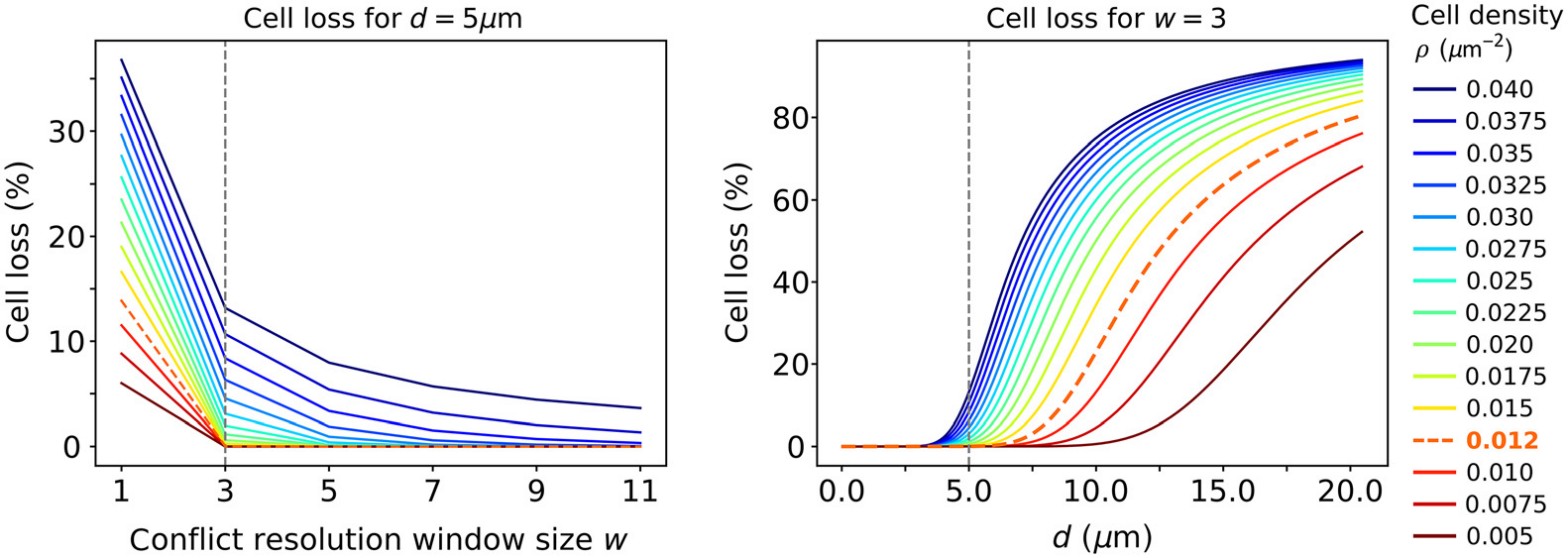
Expected cell loss for different target grid spacings d, conflict resolution window sizes w and cell densities ρ. Left: fixed target grid spacing d=5  μm and vertical dashed line denotes simplest nontrivial conflict resolution size w=3. Right: fixed conflict resolution window size w=3 and vertical dashed line denotes target grid spacing chosen for all presented experiments (5  μm). Cell density of our dataset is $\rho \approx 0.012\;{\mu m}^{-2}$ (orange dashed line).

In Fig. 2, we visualize $C_{\text{loss}}(w,d)$ for different values of w, d, and cell densities ρ.

Note that for comparison with empirical data that use an adaptive local conflict resolution window w (see Supplementary Material S.1), using w=3, provides the best approximation.

### 2.3 Step 3: Image Creation

In the last step of Cell2Grid, a low-resolution output image is created. Each node of the target grid stores the f extracted features of its assigned cell, like pixels in conventional RGB images storing three color values. This final data structure is a tensor of size $K_{x_{1}}\times K_{x_{2}}\times f$ where $K_{x_{1}}$ and $K_{x_{2}}$ are the number of GNs in $x_{1}$ and $x_{2}$ dimension. The GNs without cells are assigned zero as a default value for every cell feature. Because a square grid was used, this tensor can be converted directly into an image with f color channels, e.g., using the multipage supporting tagged image file format (TIFF). This multipage image, which we term Cell2Grid image, is the final output of the image creation step and the whole Cell2Grid algorithm (see Figs. 1 and 8 for examples).

## 3 Experiments and Results

To evaluate the utility of Cell2Grid in practice, we selected an inhouse dataset of histologic images from stage II colon cancer patients. In our experiments, we aim to investigate (a) the data processing time and cell loss of Cell2Grid for different target grid spacings, (b) how conventional cell-based features are influenced by the spatial approximation of cell locations, and (c) how CNNs perform on the task of predicting the patients 5-year tumor recurrence from histologic images using our method compared to conventional image rescaling.

### 3.1 Data for Experiments

Surgically removed tumor tissue samples from N=54 patients were collected from the local biobank affiliated to the university hospital where patients had been treated (Ethics Committee number: 28-342 ex 15/16). Each patient’s tumor recurrence status after 5 years was labeled as either relapse (17 patients) or healthy (37 patients), serving as the variable to be predicted by classification CNNs.

To obtain fm-IHC images (see Fig. 1 for an example), one formalin-fixated paraffin-embedded tumor tissue sample per patient was stained with fluorescence-conjugated antibodies targeting CD3, CD8, CD45RO, PD-L1, FoxP3, Her2, and DAPI. Of each whole-slide scan, several ROIs covering an area of 674×506  μm were selected by a human expert and were recorded with 20× magnification (1344×1008  px∼0.5  μm/px resolution) using a Vectra 3 microscope (PerkinElmer, Inc.). Spectral unmixing of raw images was performed using inForm software (Akoya Biosciences, Inc.). In total, n=1353 images (≈25 per patient) were recorded.

### 3.2 Grid Spacing, Assignment Conflicts, and Processing Time

We first investigated the empirical algorithm runtime and compression ratio of Cell2Grid and if real cell loss numbers follow our analytical expressions. Cell segmentation (step 1) was performed using inForm software (Akoya Biosciences, Inc.) with parameters set by an IHC expert. Cell nuclei were identified using DAPI stain, and cell membranes and cytoplasm were subsequently identified using remaining markers. On average, 4131 cells per image were identified (cell density $\rho =0.0121\;\mu m^{-2}$). For each cell, we extracted f=6 features using the average marker intensity across the entire cell area of each fluorescent color channel (excluding DAPI).

**Fig. 3:**
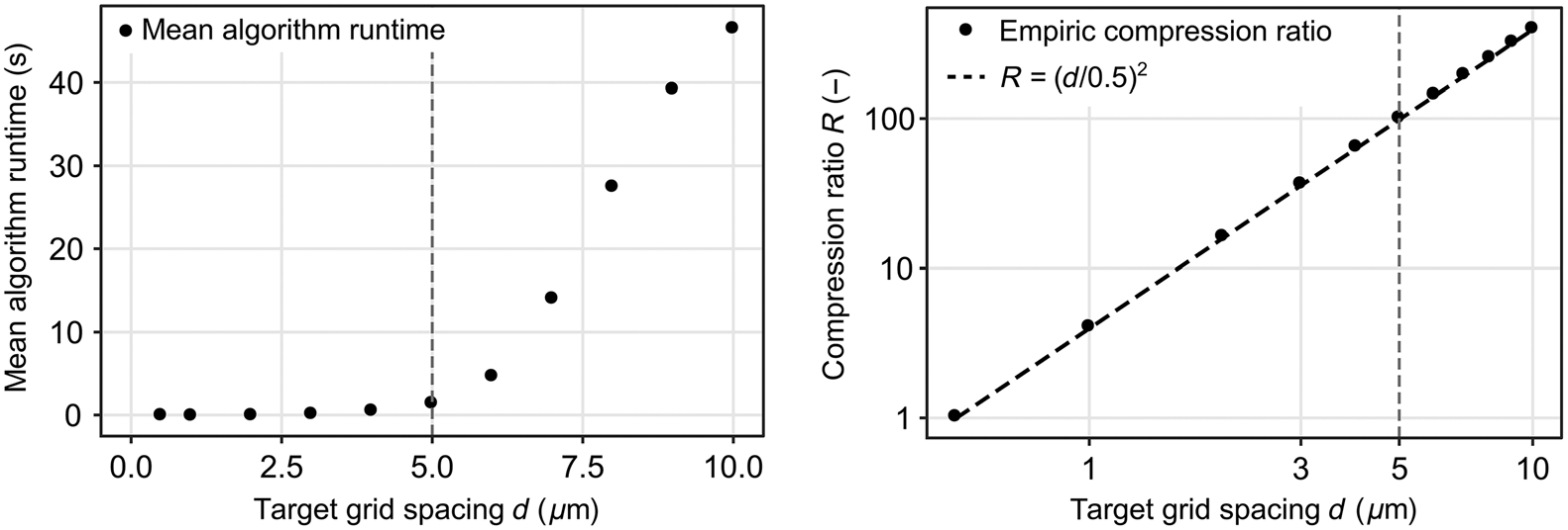
Mean algorithm runtime per image and image compression ratio (double-log plot) for different target grid spacings. Vertical dashed line represents our final choice of d=5  μm.

**Fig. 4:**
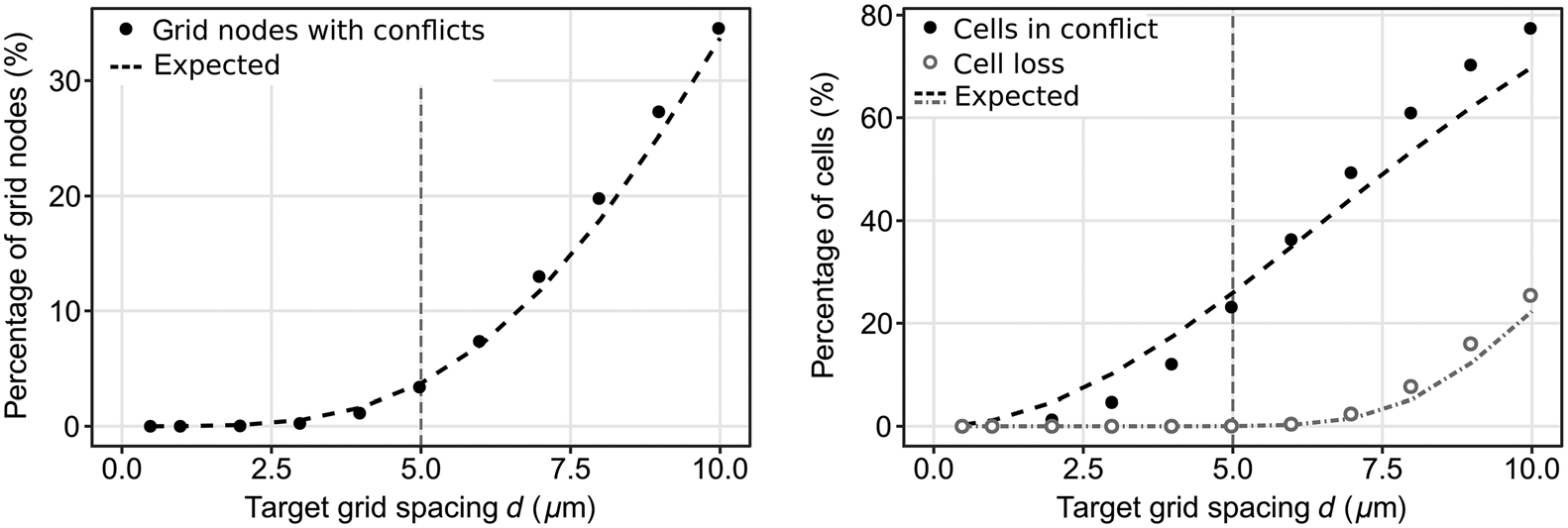
Cell assignment properties for different target grid spacings averaged over all images of our dataset. Left: percent of GNs with conflicts before conflict resolution. Right: initial percent of cells in conflict and cell loss after conflict resolution. Vertical dashed lines represent our final choice of d=5  μm.

We then investigated how different target grid spacing values influenced Cell2Grid, using a maximum local conflict resolution size $w_{\max}=7$, see Figs. 3 and 4. Algorithm runtime remained flat below d=5μm and increased steeply afterward. As expected, image compression ratio increased with the square of the target grid spacing $R={\left(d\;[\mu m]/0.5\;\mu m\right)}^{2}$ with 0.5  μm being the original image resolution.

Figure 4 shows that the percentage of GNs with conflicts and cell loss due to unresolvable conflicts followed the expected behavior [Eqs. (2), (3), and (7)]. However, the percentage of cells in conflict before conflict resolution was lower than expected below d=5  μm and higher for larger values of d due to non-uniform distributions of cells in real biological tissue. More importantly, cell loss remained neglectable below d=5  μm and increased for higher values.

Based on these findings, we decided to use d=5  μm as the target grid spacing of Cell2Grid for our following experiments. Using our dataset, this value provided a compression ratio of R=100, average cell loss of 2.06 cells (0.05%) per image, and processing time of 1.56 s per image (excluding cell segmentation).

**Fig. 5:**
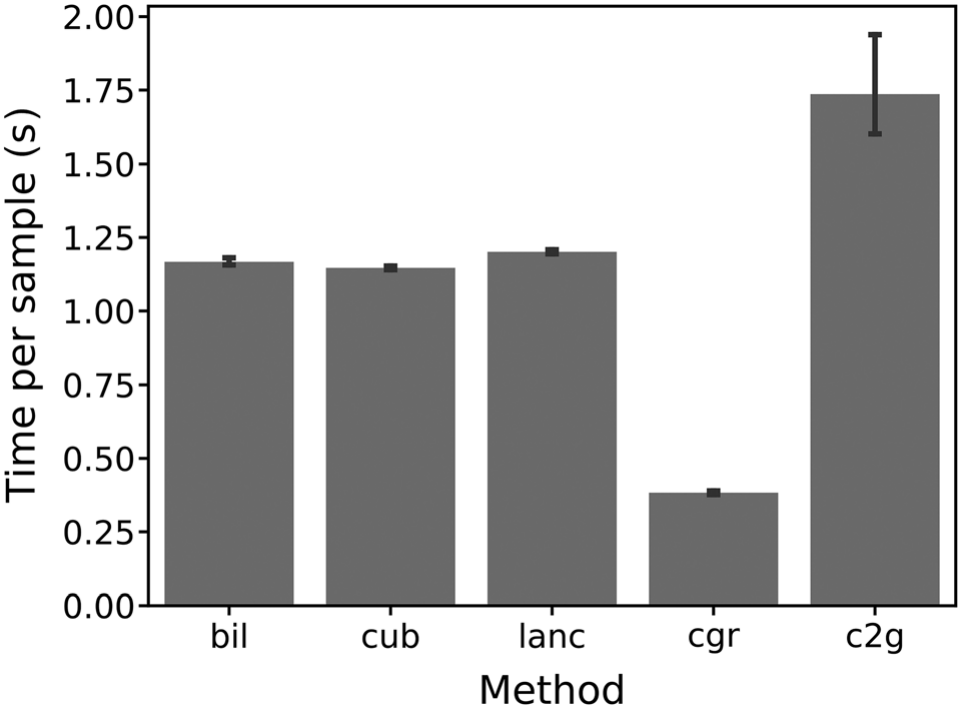
Mean and 95% confidence interval for data processing times per sample shown for image rescaling with bilinear (bil) and cubic (cub) interpolations and Lanczos (lanc) sampling, cell-graph (cgr) creation and Cell2Grid (c2g) representation, the latter two starting from cell segmentation data and do not include cell segmentation processing time. All presented times include read and write operations, multipage-TIFF-file unpacking and stacking (bil, cub, and lanc), and necessary metadata extraction.

To put the processing time of our method into perspective, we compared it with three conventional image rescaling methods and with the creation of a cell graph using cell segmentation data. Cell graphs were created using k-nearest-neighbor algorithm (k=5, see Fig. 8(c) for an example). Figure 5 shows the mean data processing time for each method but does not include time for cell segmentation required for cell graph creation and Cell2Grid. The comparably high variance in Cell2Grid is a result of the variance in cell densities and subsequent conflict resolutions per image, the latter being the main driver of computation time in Cell2Grid.

### 3.3 Phenotype Counts and Spatial Features

**Fig. 6:**
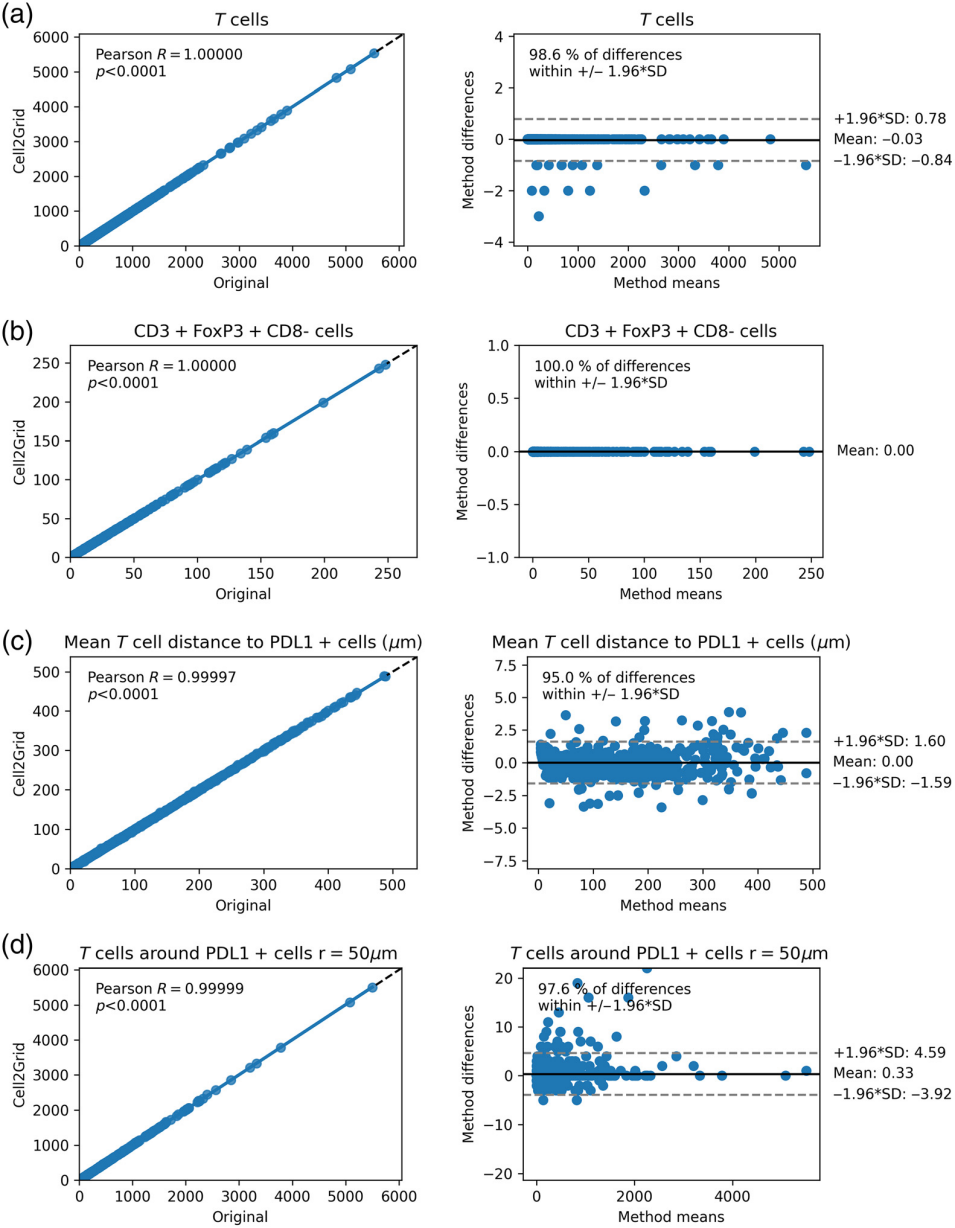
Comparison of conventional spatial features calculated using original cell segmentation coordinates versus Cell2Grid coordinates using Pearson correlation (left column, showing correlation coefficient and p-value) and the same data visualized using Bland–Altman plots (right column, showing percentage of measurements lying within an interval of ±1.96 times the SD around the mean). Top row to bottom: (a) number of T cells per image, (b) number of CD3 + FoxP3 + CD8 cells (regulatory T cells) per image (no change, SD = 0), (c) mean distance between T cells and nearest PD-L1 positive cell, and (d) count of T cells within 50-μm radius around PD-L1 positive cells.

Next, we investigated if conventional spatial features calculated from cell segmentation output tables were altered due to the local spatial approximations performed in Cell2Grid. For every individual image of our colon cancer relapse dataset, we calculated the total number of T cells (CD3+) and regulatory T cells (CD3 + FoxP3 + CD8−) to investigate the potential change in a dense and a rare cell population, respectively. Additionally, we investigated a potential change in the average distance between T cells and their nearest PD-L1 positive cell as well as the number of T cells around PD-L1 positive cells within a radius of 50  μm using the phenoptr R package.^34^ Marker thresholds for these phenotypes were set manually. Figure 6 compares these feature values calculated with original cell segmentation coordinates and coordinates as approximated by Cell2Grid. Regression plots (Pearson R>0.99, p<0.0001) showed strong agreement between both methods, which confirms that the small number of deleted cells introduces only insignificant changes in the data. Additionally, Bland–Altman plots, offering an alternative way to test the same hypothesis, show that at least 95% of the method differences fall within the predefined interval of ±1.96 times the standard deviation (SD), which is a sign of good agreement between the two measurements.^57^ In particular, no more than three T cells were deleted in any given image. Notably, no regulatory T cell was deleted in any image, indicating that rare cell populations are less likely to be affected by cell loss simply due to their lower abundance. Furthermore, the mean distance between T cells and their nearest PD-L1 positive cell was altered by <±1.6  μm for 95% of images, and the count of T cells within a radius of 50  μm around PD-L1 positive cells was altered by a count of <±5  cells in >97% of images. Results for additional features, including cell marker distributions within the cells and nucleus and cell shape properties, are shown in Supplementary Material S.4.

### 3.4 Neural Network Image Classification

Finally, we investigated how different CNN architectures performed on Cell2Grid images, and if given a fixed image size, Cell2Grid data representation improved performance compared with standard image rescaling. For the retrospective colon cancer dataset introduced in Sec. 3.1, the goal was to predict the patients’ 5-year tumor recurrence status (relapse/healthy) from histologic images in a supervised training setting. For that purpose, we trained two different CNN architectures (VGG and small interpretable network (SIN), explained below) on Cell2Grid images and on raw images rescaled with three conventional image rescaling methods (bilinear (bil) interpolation, bicubic (cub) interpolation, and Lanczos (lanc) sampling),^58^ all using a final resolution of 5  μm/px.

To investigate the effects of additional cell shape features in Cell2Grid, we also trained a CNN on Cell2Grid images that contain, apart from the six default mean marker channels, three additional image channels describing the cell shapes (nucleus size, cell size, and cell axis ratio). We refer to these image set as c2gShape. Additionally, we trained a GNN on a cell graph created from the same cell segmentation data used for Cell2Grid.

**Fig. 7:**
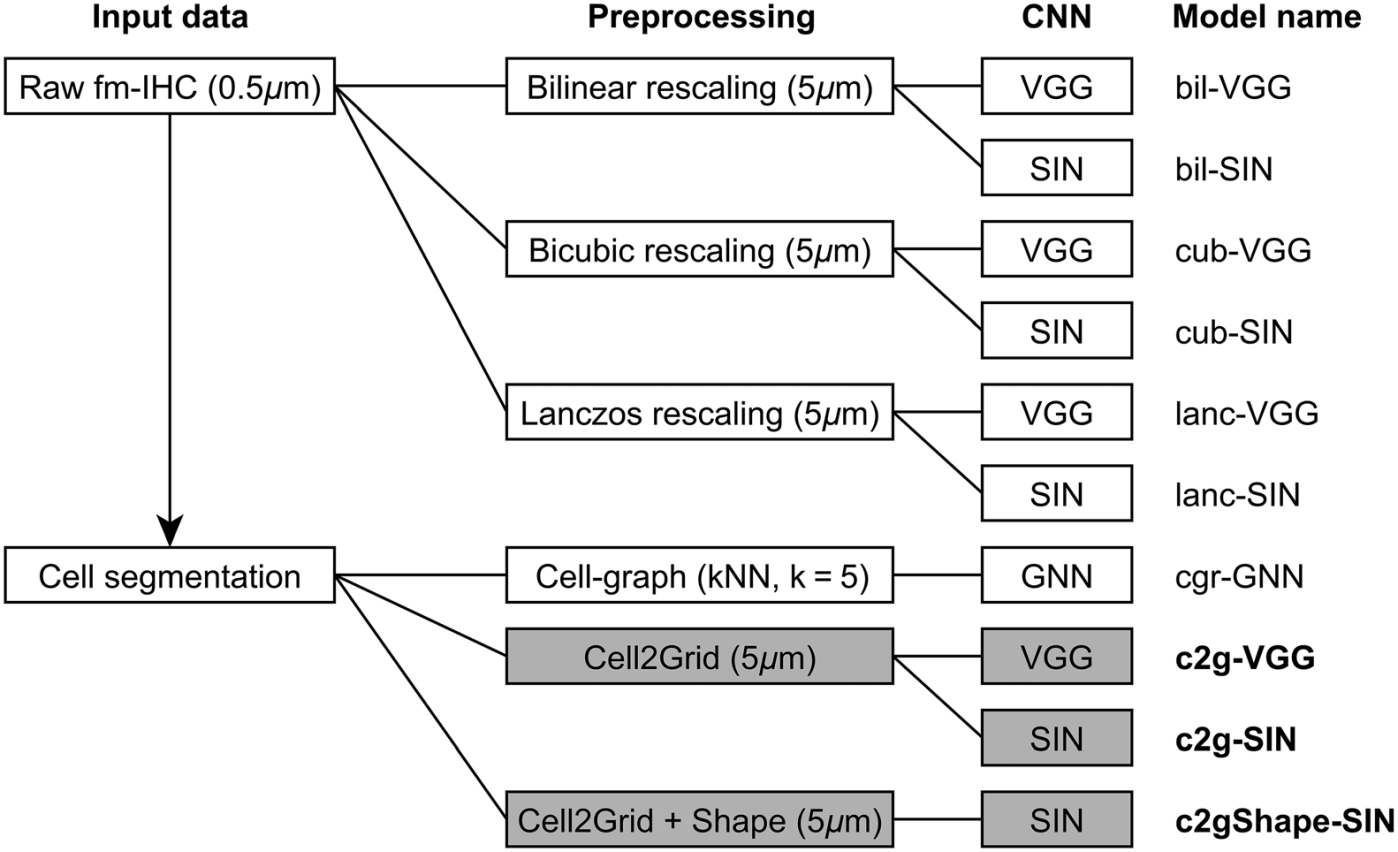
Model configurations used in our experiment. Models using our method highlighted in gray.

**Fig. 8:**
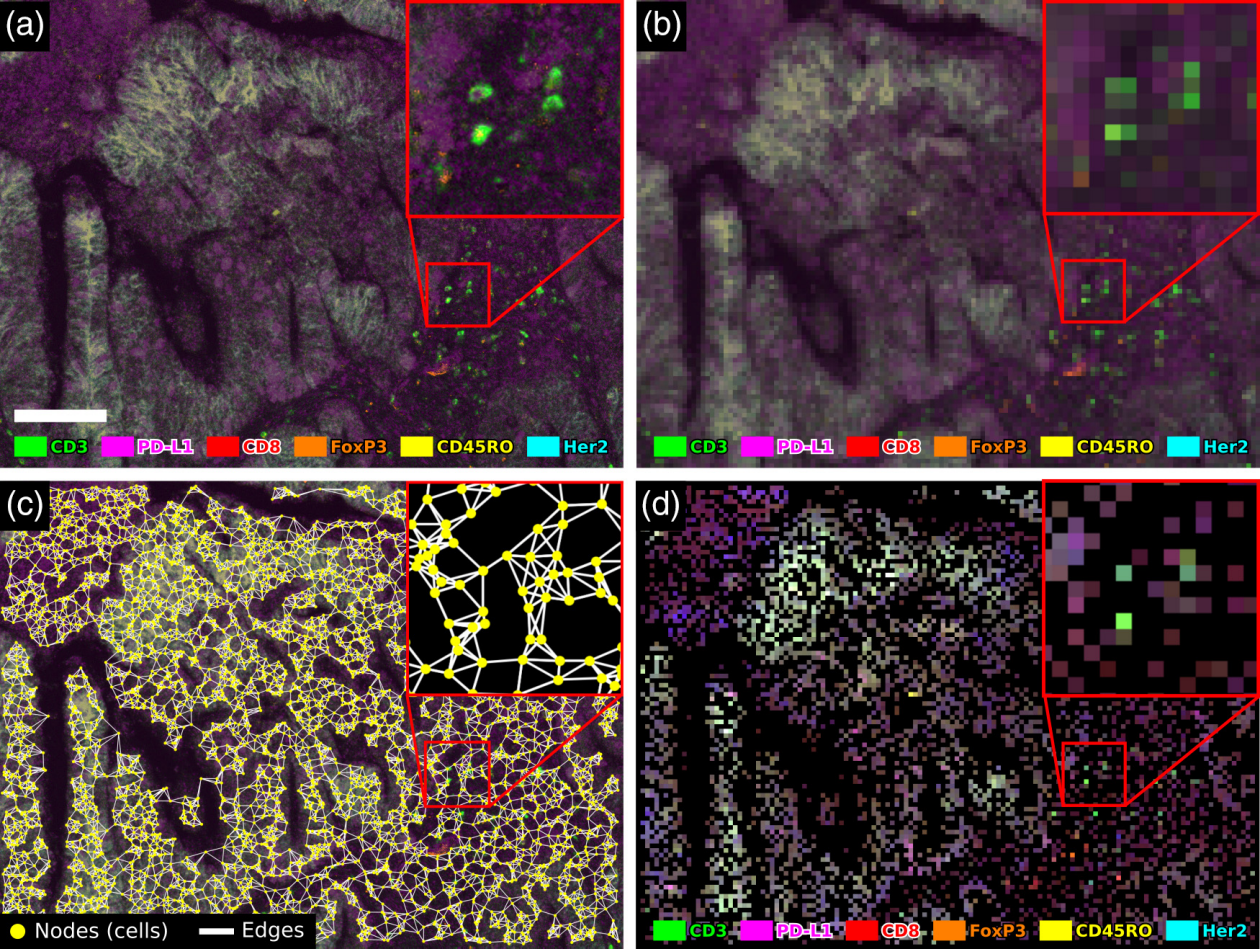
Comparison of image representation methods using false color images of six marker channels. Scale bar 100  μm. (a) Original fm-IHC (0.5  μm resolution), (b) conventional bil rescaling (5  μm), (c) cell segmentation output (yellow dots) and cgr (white lines), and (d) Cell2Grid image (5  μm target grid) using the same cell segmentation data as shown in panel (c). Image best viewed on screen.

Figure 7 shows the entire experiment setup, and a side-by-side comparison of all data modalities is shown in Fig. 8. A description of interpolation-free data augmentation schemes required for Cell2Grid images is presented in Supplementary Material S.5.

#### 3.4.1 Modified VGG-16 architecture

As a baseline for CNN image classification, we chose the well-established VGG-16 architecture,^3^ which, despite its age and simplicity, has been used frequently with biological tissue images in recent research.^59^^–^^64^ To suit the six-color-channel image data format of our images, we modified the architecture using a different first convolutional layer. Fully connected layers at the top of the model varied in number of neurons compared with the original VGG-16 due to the change in input image size and output dimensions. We simply refer to this modified architecture as VGG in the following. This model has $>5.6\cdot 10^{7}$ parameters, all of which were initialized randomly. Results for model runs with network weights pretrained on the ImageNet dataset^6^^,^^65^^,^^66^ are shown in Supplementary Material S.6.

#### 3.4.2 Custom network architecture SIN

As a second model, we use a custom CNN architecture in the style of VGG-16 that uses fewer layers and parameters. We refer to this architecture as SIN in the following. For the first layer of SIN, we used a convolution with a 1×1 kernel inspired by the network-in-network approach of Lin et al.^67^ The weights of this layer were subjected to L1-regularization with regularization factor $\lambda =10^{-4}$. This was followed by 4 pairs of 3×3 convolutions with 16 feature maps each and subsequent max pooling layers. On top of the network, we placed 1 fully connected layer with 16 nodes, followed by a dropout layer (33 %) and a final SoftMax layer with 2 outputs. This architecture uses $<10^{4}$ trainable parameters and has at most 16 feature maps per layer. No pretraining was applied to this model. Table 2 SIN architecture summarizes the SIN architecture.

**Table 2:** SIN architecture.

Layer type	Output Shape	Params #
InputLayer	(102, 135, and 6)	0
Conv2D	(102, 135, and 16)	96
Conv2D	(102, 135, and 16)	2304
MaxPooling2D	(34, 45, and 16)	0
Conv2D	(34, 45, and 16)	2304
MaxPooling2D	(11, 15, and 16)	0
Conv2D	(11, 15, and 16)	2304
MaxPooling2D	(3, 5, and 16)	0
Conv2D	(3, 5, and 16)	2304
MaxPooling2D	(1, 1, and 16)	0
Flatten	(16)	0
Dense	(16)	272
Dropout	(16)	0
Dense	(2)	34
Total params: 9618		
Trainable params: 9618		
Nontrainable params: 0		

#### 3.4.3 Graph neural network

As a reference, we compared all methods with GNN trained on cell graphs using the same cell segmentation data that were used for Cell2Grid. We created cell graphs from cell segmentation data using k-nearest neighbors (k=5) and an edge cutoff length of 25  μm following Jaume et al.^46^ (see Fig. 8(c) for an example). Each node was described with the same cell features as in Cell2Grid data. We used the best GNN architecture for graph classification as identified by You et al^48^ implemented using the Spektral Python package.^68^

#### 3.4.4 Training setup

From the total of n=1353 recorded images, we used the same hold-out test set of 247 images (176 healthy and 71 relapse) for all models. The remaining 1106 images were randomly split into a training and validation set (70 %/30 %) stratified by class label using the same split for each model. Oversampling of the minority class was used during training to account for class imbalance. We used a class weight of 3:1 in favor of the relapse class to emphasize the clinical importance of predicting relapse cases. Early stopping was used to end training when validation set error rate stopped decreasing. We used a batch size of 64 and binary cross-entropy as loss function and the Adam optimizer.^69^ Learning rate and layer initialization of VGG were subjected to hyperparameter optimization (see Supplementary Material S.6).

All models were trained on an off-the-shelf desktop workstation with a single GPU (Nvidia RTX 2070). Models were implemented in Keras^65^ with a TensorFlow backend.^70^ We trained each model ten times and evaluated the prediction performance of each run on the validation and test set using the balanced error rate, a metric equivalent to the mean of the false-positive and false-negative rate of model predictions.

#### 3.4.5 Results

**Fig. 9:**
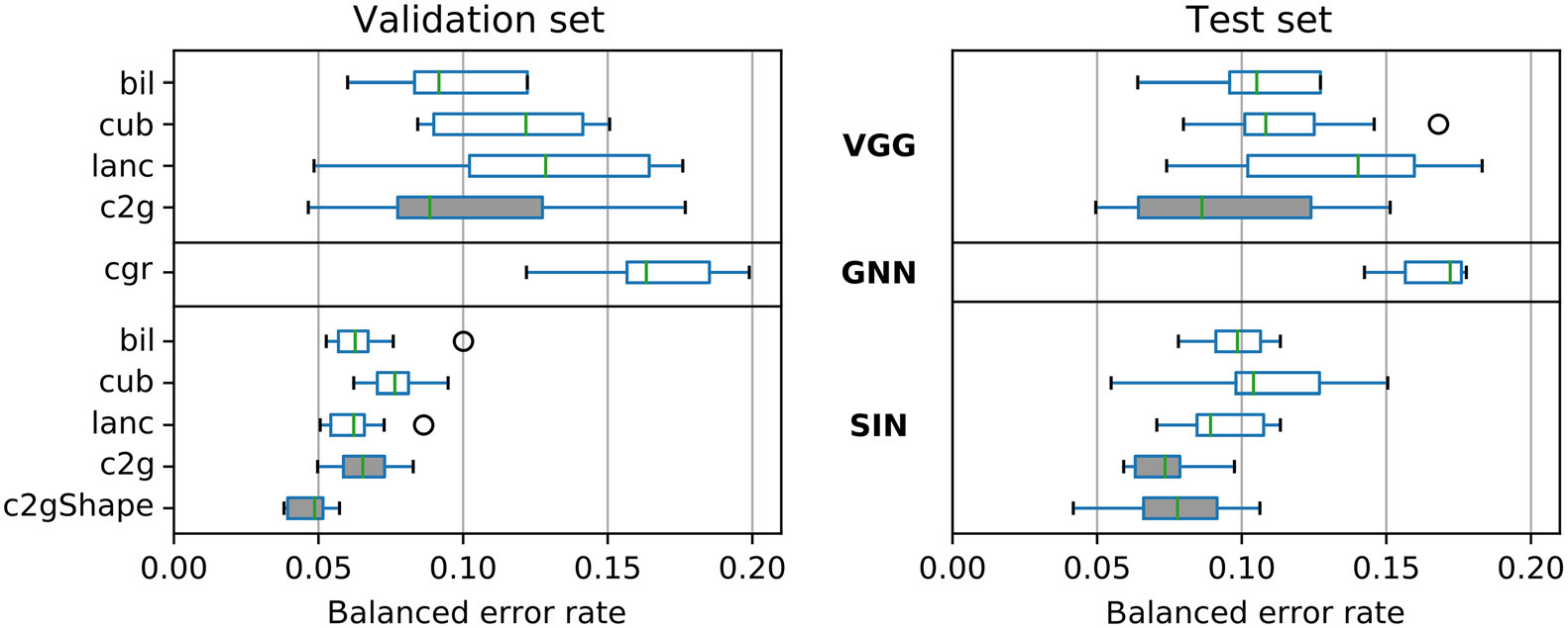
Balanced validation and test set error rates for all models (lower is better), ten repeated runs summarized by a single boxplot each (two outliers for bil-VGG and one for Cell2Grid-VGG with error rates above 0.2 not shown for clarity, see Table 3). Datasets indicated on the left and network architectures in the middle in bold. Models trained with our method (Cell2Grid and c2gShape) are highlighted in gray.

**Fig. 10:**
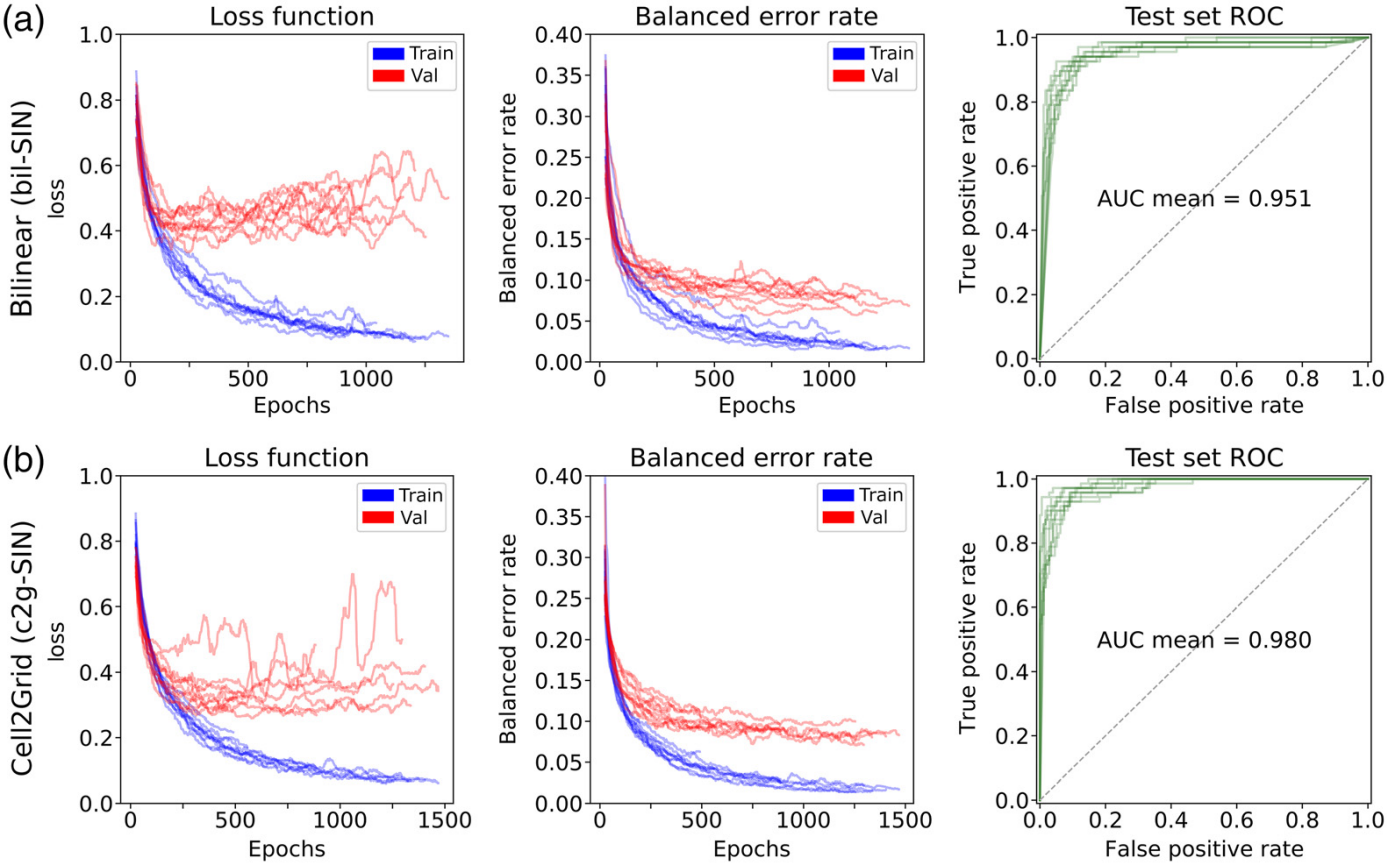
Smoothed training curves for loss function and balanced error rate as well as test set ROC curve with mean area under the curve for all ten SIN model runs. (a) SIN trained on bil rescaled images (bil-SIN) and (b) SIN trained on Cell2Grid images (c2g-SIN).

To assess model performance, we report the balanced classification error rate ${\mathrm{err}}_{\mathrm{bal}}=1-{\mathrm{acc}}_{\mathrm{bal}}$ calculated from the balanced image classification accuracy ${\mathrm{acc}}_{\mathrm{bal}}$ of model predictions compared with the known ground truth label (5-year tumor recurrence status). Results of all models and ten repeated runs each are summarized in Table 3 and Fig. 9. VGG models showed higher variance in error rates compared with SIN, regardless of image processing method. As shown in Supplementary Material S.6, different training settings for VGG did not improve its performance. We found that conventional rescaling methods bil and cub resulted in models with comparable performance on the test set with lanc falling slightly behind. Despite similar validation set error rates, Cell2Grid models maintained lower test set error rates compared with bil, cub, and lanc models, especially for the SIN architecture, indicating better generalization properties and less overfitting. Figure 10 shows a comparison of the training curves of the Cell2Grid-SIN and bil-SIN models and test set receiver operating characteristic (ROC) curves for all ten repeated model runs.

**Table 3:** Balanced error rates for all models and ten repeated runs. See Fig. 9 for a visual representation.

Model	Balanced error rate median (min–max)		Relative test error rate compared with bil model (lower is better)	Relative improvement compared with bil model
	Validation set	Test set		
bil-VGG	0.092(0.060–0.500)	0.105(0.064–0.500)	1.000	—
cub-VGG	0.121(0.084–0.151)	0.108(0.080–0.168)	1.028	—
lanc-VGG	0.140(0.102–0.176)	0.152(0.102–0.183)	1.448	—
Cell2Grid-VGG	0.088(0.046–0.296)	0.086(0.050–0.297)	0.819	18.1%
cgr-GNN	0.163(0.122–0.200)	0.172(0.143–0.178)	—	—
bil-SIN	0.063(0.053–0.100)	0.099(0.078–0.113)	1.000	—
cub-SIN	0.076(0.062–0.095)	0.104(0.055–0.151)	1.051	—
lanc-SIN	0.062(0.050–0.086)	0.089(0.071–0.113)	0.899	10.1%
Cell2Grid-SIN	0.065(0.050–0.083)	0.074(0.059–0.097)	0.748	25.3%
c2gShape-SIN	0.049(0.038–0.057)	0.078(0.042–0.106)	0.788	21.2%

Additional cell shape features, as used in c2gShape-SIN, led to the best validation set error rates, but did not improve upon Cell2Grid-SIN (which use only fluorescent marker features) on the test set across multiple model runs. We attribute this to c2gShape containing more information but also being more prone to overfitting due to its additional input features. Nevertheless, the single best performing model run was a c2gShape-SIN model (3.8% and 4.2% balanced error rate on the validation and test set, respectively). The GNN model used in our experiment showed higher test set error rates than all CNN models. As shown in Table 3 and Fig. 9, models trained on Cell2Grid data led to an 18.1% and 25.3% relative reduction of test set error rate for VGG and SIN models, respectively, compared with conventional bil rescaling.

As an additional experiment, we investigated if higher input image resolution in the bil-SIN models could improve model performance compared Cell2Grid-SIN. We therefore rescaled our data to 2.25 and 1.0  μm/px (images sizes 300×255  px and 675×506  px, respectively) using bil and used the same training setup as before.

**Fig. 11:**
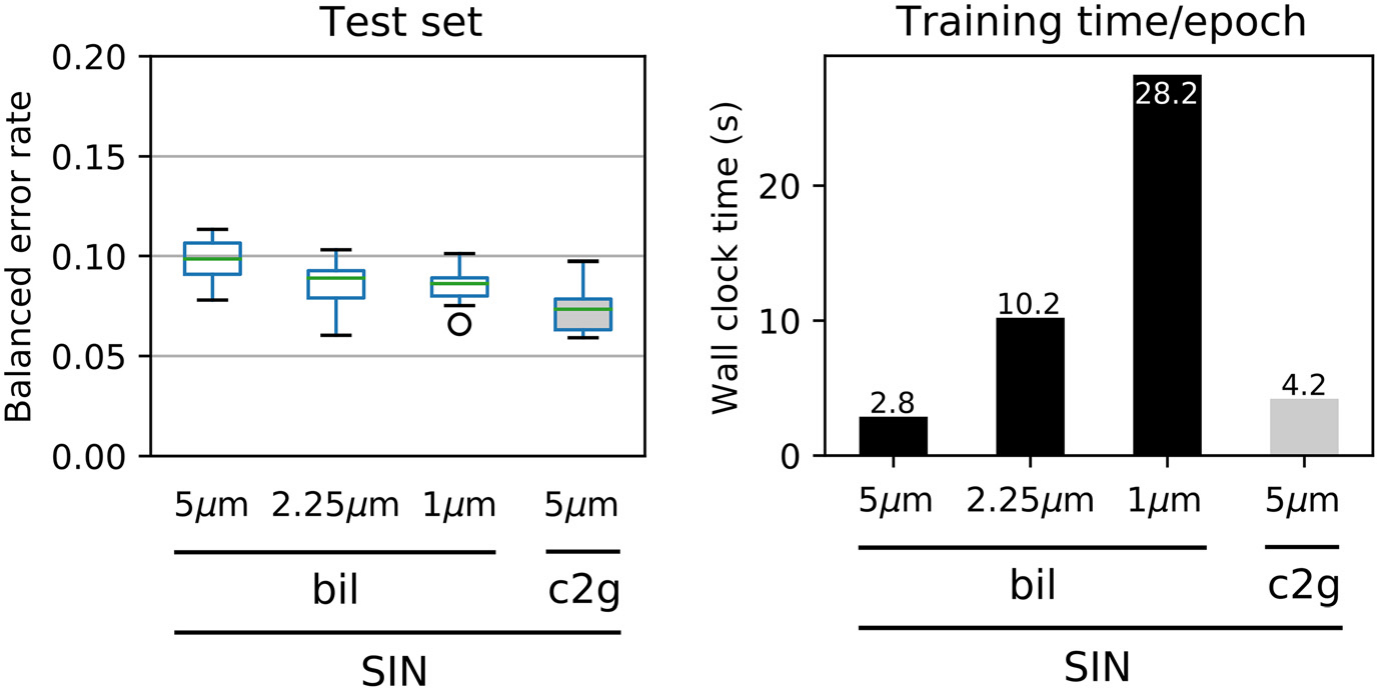
Balanced test set error rates and per-epoch training times for bil-SIN models at different resolutions (5, 2.25, and 1  μm) compared with Cell2Grid-SIN (5  μm). Our method highlighted in gray.

Figure 11 shows that test set error rates of bil-SIN decreased with increased image resolution, but remained above Cell2Grid-SIN, even at 1  μm/px. However, the larger images sizes at higher resolutions led to longer training times due to the increased number of network parameters in the top layers and slower data augmentation. Specifically, per-epoch training time of Cell2Grid-SIN at 5  μm was decreased by 85% compared with bil-SIN at 1  μm while simultaneously achieving lower test set error rates.

## 4 Discussion

CNN architectures have become increasingly complex, requiring long training times and powerful hardware, particularly when using large images for training.^1^^–^^3^^,^^5^ In some domains, rescaling images to a much lower resolution still allows for efficient image classification. In the biomedical domain, however, this is rarely applicable, as often not only the whole picture but small details can be crucial for classification, such images containing cells. In this paper, we presented an alternative to rescaling that allows for efficient training of CNNs without altering the essential morphological properties of these images.

The Cell2Grid images are different from conventional images in a number of ways: (1) they contain no natural gradients, because every pixel in the image is an independent object (a biological cell); (2) they might contain many empty pixels (numerically zero) in areas without assigned cells; and (3) they cannot be rotated, zoomed, sheared, or transformed for data augmentation with methods that include pixel interpolation because the value and integrity of individual pixels matter (see Supplementary Material S.5). As shown in Fig. 8(d), final Cell2Grid images are sparse, containing several empty (black) pixels. In our example, Cell2Grid images (at 5  μm/px) had ten times fewer pixels in each spatial dimension compared to the original fm-IHC images (0.5  μm/px), corresponding to an image-to-image compression ratio of 100. In contrast, in conventional image downscaling methods, pixels that belong to different biological cells may be used to obtain a pixel value of the new target resolution through interpolation. This makes it difficult or sometimes even impossible to identify individual cells and their true marker expression in strongly downscaled images [Fig. 8(b)]. Due to the cell segmentation step prior to Cell2Grid that uses the original high-resolution images, the concept of individual cells is retained in its output and the cell features, as extracted during cell segmentation, are left unaltered. Keeping cell information intact is key in many applications, such when studying colon cancer,^71^ where the presence of just a few cells with distinct phenotype has been shown to be an important image feature.

In our case study, we mainly focused on the average fluorescent marker intensities as features for each cell as this leads to images that are most similar to conventionally rescaled images. Depending on the scientific question, other features may be relevant, including size and shape of nuclei and cells and marker heterogeneity within the cells. Our experiment on the inclusion of cell shape and size properties as additional image color channels revealed that these features may contribute to better performing models but tend to be more prone to overfitting and therefore need to be used with care. Using additional image color channels, including marker distribution properties (see Supplementary Material S.4) may be used in the same fashion.

We used a SIN with $<10^{4}$ parameters, which is orders of magnitude smaller than our modified VGG architecture with $5.6\cdot 10^{7}$ parameters. We found that the smaller SIN model more stable during training compared with VGG and performed, on average, better than VGG on unseen test samples. As smaller networks require less GPU memory, they allow for larger batch sizes or larger input images during training. This is particularly relevant toward training on whole-slide images to capture the entire tissue heterogeneity, a feature commonly observed in tumors.^72^

For evaluation, we compared Cell2Grid with conventional image rescaling with bil and cub interpolations as well as lanc sampling. Instead of merging pixels based on their proximity to the pixels of the target resolution, our method merges only pixels that belong to the same biological cell, which is a process related to the concept of superpixels.^73^^,^^74^ This cell-based approach of Cell2Grid enables subsequent analysis, such as cell counting, phenotyping, and cell-based interpretation of a trained neural network.^75^^,^^76^ Graph-based methods, including GNNs^45^^,^^76^^–^^78^ also rely on the output of cell segmentation or nuclei identification in histologic images but they require a cell graph-based on the segmented cells and their coordinates. Our method follows a different approach by converting the list of segmented cells into a low-resolution image suited for training a CNN. While the input data for our method is the same as for cell graph creation, the output of Cell2Grid allows for the training of conventional, well-established CNNs, as compared with the more recent GNN architectures.

In the last decade, higher standards for privacy protection complicated the sharing of data obtained from human samples. The Cell2Grid provides a way to share pathology data in a compact format that does not contain the original high-resolution histologic image. We therefore hope that this technique reduces concerns for data protection and encourages researchers to release datasets in this format.

### 4.1 Limitations and Future Work

The research scope of this work focused on the investigation of an alternative data representation of high-resolution histologic images to facilitate their use with CNNs. Therefore, the first step of our algorithm directly relied on the output provided by commercial software available in most laboratories and provided a standardized scenario on which to experiment. Moreover, in our current implementation, an IHC expert had to choose which method and parameters were best suited for the biological tissue. Because our method strongly depends on this cell segmentation step, this set of constraints constitutes an important limitation. Our research scope did not include an investigation of the effects of different cell segmentation algorithms, but fully automated cell segmentation is an active field of research^24^^,^^30^^,^^31^^,^^53^^,^^54^ that we intend to explore in future work with recently published methods showing promising results.^30^^,^^31^

Additionally, Cell2Grid requires additional processing time compared to conventional image rescaling. As shown in Sec. 3.2, data preprocessing using Cell2Grid is slower than conventional image rescaling even without accounting for cell segmentation time. Furthermore, cell graph creation was significantly faster than our method, both starting from cell segmentation data. This additional data processing time needs to be weighed against any potential gains of our method for downstream applications, like a potential reduction in CNN training time or classification accuracy.

In our experiments, we focused on a small set of cell features to construct Cell2Grid images, i.e., the mean marker expression within each cell. This enables a direct comparison with conventional image rescaling methods as the number and type of image channels remains the same. To add additional information to Cell2Grid images, other features, such as marker distribution properties across the cell and cell shape features, may be used as additional image channels in Cell2Grid output, as demonstrated by our experiments (see results for c2gShape and Sec. S.4 in the Supplementary Material). However, extracellular information or cell-based information that is either not extracted during cell segmentation or is not used as a dedicated image channel in Cell2Grid is inevitably lost from the data. This is a limiting factor for our method, especially for applications where a pathologist—or an image classification model—requires this information for accurate assessment of the images.

Regarding the assignment of cells to the target grid (step 2 of Cell2Grid), care needs to be taken, as a large target grid spacing d may lead to deletion of individual cells. Even if in small numbers, cell deletion may be undesired if tissue areas with high cell density are of particular interest, e.g., immune cell clusters in insulitis.^79^ In this case, a smaller target grid spacing d, a larger local conflict resolution size $w_{\max}$, or both are required, potentially increasing processing time. In our method, the precise location of individual cells is approximated to fit the target grid during cell assignment. However, because cells are moving in live tissue, their exact position in the final tissue section depends on the time of sampling as well as the location and angle of tissue sectioning. We estimate that the net effect of these factors is greater than the local shift in cell positions introduced by Cell2Grid, which for most cells is smaller than the target grid spacing d.

Even though we demonstrated the benefits of Cell2Grid using fm-IHC images of size 674×506  μm, a WSI of a tissue section can be up to 30 times bigger in each spatial dimension. In our experiments, we did not yet investigate the utility of Cell2Grid when applied to WSI data. Additionally, we only considered image rescaling instead of image compression methods (such as JPEG2000^80^) as reference data processing methods CNN training, because the latter only reduce image storage size and not memory size once decompressed and loaded into memory for model training.^8^

Our study is limited using only a single fm-IHC dataset of colorectal cancer patients that comprises only 54 patients and 1,353 images. These numbers are small compared with the large number of CNN parameters ($10^{7}$ and $10^{4}$ for VGG and SIN architectures, respectively). For a more comprehensive analysis of our method, future experiments on different datasets with larger cohorts are required to identify potential weaknesses and demonstrate robustness of our algorithm for different tissue types. Additionally, the utility of our method when applied to other image modalities, such as conventional H&E-stained images, needs to be investigated in future work.

In addition, we want to build on our current work and develop further two areas of interest.

1. Volumetric images: when studying tumor samples, even whole-slide scans of two-dimensional tissue sections may not be enough to fully capture the heterogeneity inherent to colorectal cancer.^72^ In this case, multiple consecutive sections of a tissue sample can be acquired with the distance between two sections being typically 3−4  μm.^81^ By setting the target grid spacing d of Cell2Grid equal to the section spacing, an isotropic 3D model of the tissue can be generated from consecutive tissue sections, such that each cell is represented by a single voxel. These volumetric images can be used to train 3D CNNs^82^ that capture the entire structure of a tissue sample. A showcase using consecutive sections of a colon cancer sample is currently being developed.
2. Synthetic image creation: in medicine, an important goal of using machine learning tools is the extraction of knowledge from a trained model. As summarized by Jaume et al.,^76^ model explanations that operate on a pixel-level suffer from real interpretability by not taking into account that the important entities of a histologic image are the individual biological cells and their spatial distribution.^83^^,^^84^ In a first use-case of murine pancreatic islets, we showed how to exploit the cell-based data representation of Cell2Grid to algorithmically create synthetic image datasets with known ground truth using a procedural algorithm for the creation of Cell2Grid images and a cGAN to turn Cell2Grid images into original fm-IHC images.^85^ This will support the creation of a pipeline for knowledge extraction from CNNs^75^^,^^86^^–^^89^ trained on Cell2Grid images.

## 5 Conclusions

In this paper, we introduced Cell2Grid, which is an algorithm for efficient representation of histologic images whose output can be used directly to train CNNs. As a case study for evaluation, we used fm-IHC images to predict 5-year relapse risk of stage II colon cancer patients. Our results showed that Cell2Grid preserves small-scale cell level information and how this information can reduce the test set error rate of a neural network by 25% relative to conventional bil rescaling at the same resolution while also reducing training time by 85% compared with higher resolution reference models. However, future experiments are required to investigate the performance of our method when applied to other image types, such as H&E-stained images and conventional chromogenic IHC.

Compared with conventional image rescaling methods, images transformed with Cell2Grid are directly suited for cell-based analysis, and in addition, they might simplify interpretation of trained CNNs. The Cell2Grid therefore opens a door for further exploring the use of histologic images with deep learning architectures and in a wider field of applications, such as volumetric images and synthetic image creation.
