# Supplementary material for: Cell2Grid: an efficient, spatial, and convolutional neural network-ready representation of cell segmentation data
Source: J Med Imaging (Bellingham). 2022 Nov 30;9(6):067501. doi: 10.1117/1.JMI.9.6.067501 (PMC9709305; doi:10.1117/1.JMI.9.6.067501)
Supplement: Supplementary file 1 [file JMI_009_067501_SD001.pdf]

# S. Supplemental Material

## S.1 Resolving assignment conflicts

This section provides additional details on the conflict resolution method (step 2) of Cell2Grid.

### S.1.1 Munkres' algorithm

Munkres' algorithm[55], also known as the *Hungarian algorithm*, provides a solution to the *linear assignment problem*: given $N$ workers and $M$ tasks, it finds the cheapest way to solve as many tasks as possible, under the condition that each worker can only complete one task, and each task requires exactly one worker to complete[90]. The cost $c_{ij}$ for worker $i$ to complete task $j$ may be different for each worker-task combination. We denote these individual costs with the cost matrix $C_{N\times M}$, which is square for $N = M$. Finding an optimal solution can be expressed as changing the order of rows and columns in $C$ until the sum of diagonal elements is minimal, i.e. $\arg\min_{L,R} tr[LCR]$ , where $L$ and $R$ are matrices denoting the permutations of lines and rows. The diagonal elements of $C$ then represent the worker assignments.

This problem is identical to the cell assignment during conflict resolution in Cell2Grid, considering that $N$ conflicting cells need to be assigned to $M$ available grid nodes. Intuitively, the cost $c_{ij}$ of assigning cell $i$ to grid node $j$ is simply a measure of the distance between $i$ and $j$. Using Munkres' algorithm to minimize $tr[LCR]$ provides the cell assignment with the least total cost, i.e., the minimal total travel distance of all involved cells.

Without modification, Munkres' algorithm can solve such an assignment problem when $C$ is square[91], i.e. $N = M$. For the cases $N < M$ and $N > M$, we can pad $C$ with rows or columns with zeros to make it square. In the case of too many grid nodes ($N < M$), this is equal to adding $M - N$ virtual cells that have 0 travel distance to any node. These cells are effectively assigned to the grid nodes that are far away from real cells. In the case of too many cells ($N > M$), padding $C$ with zeros is equal to adding $N - M$ virtual grid nodes that all cells have 0 travel distance to. Cells that get assigned to any of those virtual nodes will be deleted after conflict resolution since there were not enough real grid nodes available.

Ideally, the total assignment problem of all cells in an image is solved by a single use of Munkres' algorithm. However, the runtime of Munkres' algorithm[92] increases as $O(N^3)$ with the size of the cost matrix $N$. Computation time of several hours for a single image therefore becomes impractical. Instead, we follow the approach outlined in the main text, by first assigning all cells to their closest grid and subsequently solving assignment conflicts locally *one grid node at a time*, using Munkres' algorithm.

### S.1.2 Local conflict resolution

After initial binning of cells to their closest grid node, grid nodes with conflicts are resolved sequentially in random order. At each node, we use Munkres' algorithm to find an optimal, local assignment of *all cells* within a small *conflict resolution window* $w_{max}$ around the grid node (Figure S-1).

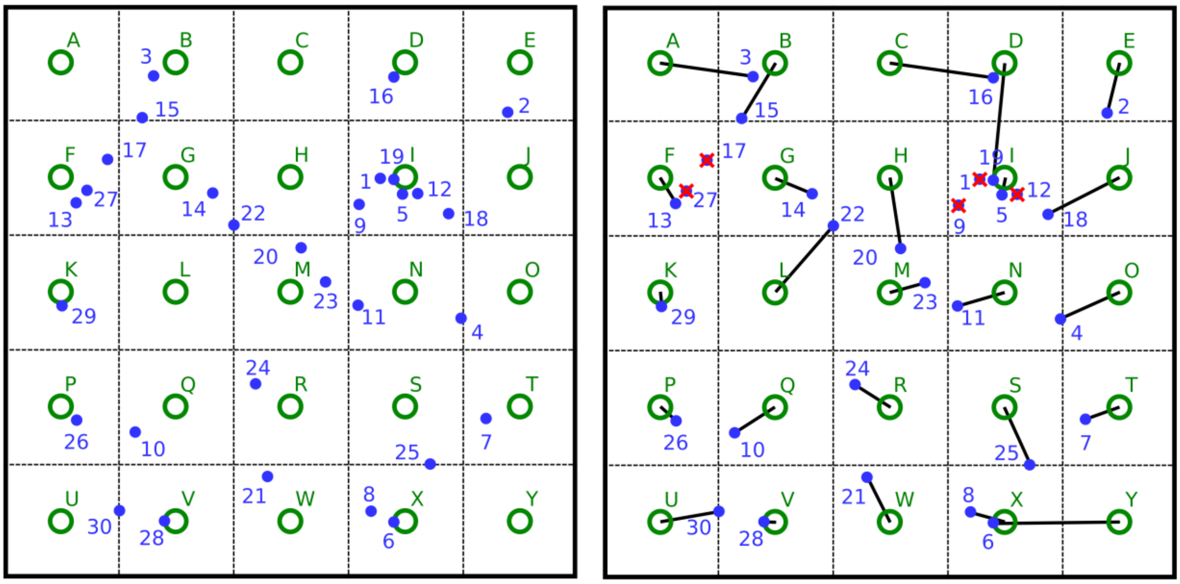


Figure S-1: Illustration of resolving assignment conflicts with $w_{max} = 5$. Left: 25 grid nodes (green circles, letters), and 30 biological cells (blue dots, numbered). The two conflicting cells 20, 23 at the central grid node M triggered the conflict resolution. Right: Solution to the assignment problem. The local 3x3 neighborhood around M contains more cells (15) than grid nodes (9), the problem is therefore solved in the 5x5 neighborhood instead. Black lines indicate final cell assignment, red crosses indicate deleted cells.

For large $w_{max}$, computation time may be unnecessarily high in cases where the conflict could be resolved in a smaller local neighborhood. For each conflicting grid node we therefore start by considering the $3 \times 3$ local neighborhood around the conflict. If the number of cells $N$ within this local neighborhood is smaller or equal than the total number of grid nodes $M$ in this window ($N \leq M$) we use Munkres' algorithm to optimize the assignment of *all* cells (using their original locations) to all available grid nodes within this window. If this window contains too many cells, $N > M$, we increase the local neighborhood size to the next symmetric window ($5 \times 5, 7 \times 7$ etc.). We continue until a window size with $N \leq M$ is found or until the maximum window size $w_{max} \times w_{max}$ is reached. The entire procedure is outlined as pseudo code in the following. We implemented this method using the python package *munkres*, version 1.1.4.

**Pseudo code for cell assignment and conflict resolution with predefined $w_{max}$**

```
bin all cell coordinates to target grid
conf_list = list of all grid nodes with conflicts
for each grid node g in conf_list do:
  w = 3
  subgrid = w × w around g
  while w ≤ w_max do:
    N = number of cells within subgrid
    if (N ≤ w^2) or (w == w_max) do:
      apply Munkres' algorithm to all N cells in subgrid
      update conf_list
    else:
      w = w + 2
```

### S.1.3 Choosing a cost function

Munkres' algorithm solves assignment problems defined by a cost matrix $C$. We define its elements $c_{ij}$ using the *squared* Euclidean distance between cell $i$ to grid node $j$. Here, we provide an example that illustrates how this choice minimizes local distortions compared to conventional Euclidean distances.

Using conventional Euclidean distances, Munkres' algorithm minimizes the sum of individual travel distances of all cells, occasionally creating solutions that include unnecessary large distances of one or more cells. Using the squared Euclidean distance makes long individual travel distances increasingly expensive, effectively producing solutions for which each cell gets assigned to grid nodes in its local neighborhood. This is illustrated in Figure S-2, showing an example of two free grid nodes A and B and two cells 1 and 2 (for simplicity we assume a grid spacing $d = 1$ and that no other grid cells are available). This assignment problem has only two solutions: $[1 \rightarrow B, 2 \rightarrow A]$ (solution 1) and $[1 \rightarrow A, 2 \rightarrow B]$ (solution 2).

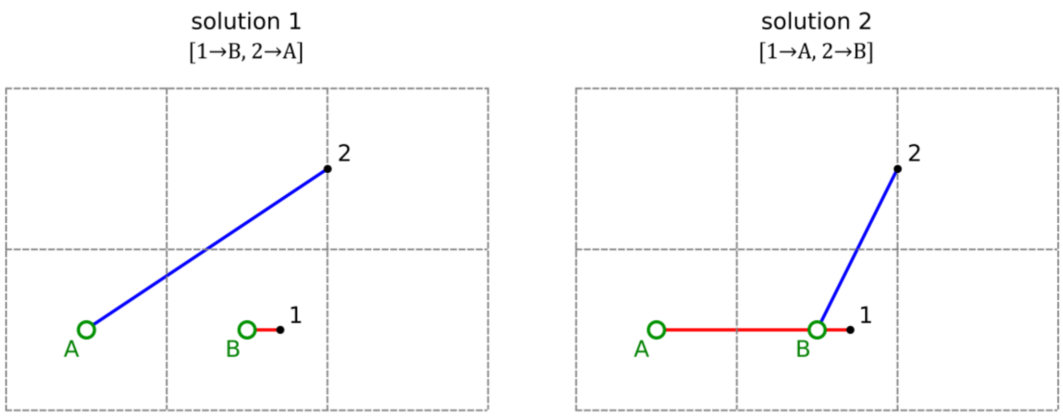


Figure S-2: Two possible solutions to an assignment problem with two available grid nodes (A, B, green dots) and two cells to be assigned to them (1, 2, black dots). Solutions visualized with blue and red lines.

While both solutions are valid, solution 1 involves a relatively long assignment distance for cell 2. Additionally, this solution swaps the location of cells along the x-axis, creating an unnecessary local distortion of the cell arrangement. In contrast, total Euclidean travel distance of solution 2 is higher, but split more equally between the two cells.

Calculating the total assignment cost of both solutions using the conventional Euclidean distance (ED) shows that the cost of solution 1 $ED[1 \rightarrow B, 2 \rightarrow A] = \sqrt{0.2^2} + \sqrt{1.5^2 + 1^2} = 2.003$ is smaller than that of solution 2 $ED[1 \rightarrow A, 2 \rightarrow B] = \sqrt{1.2^2} + \sqrt{0.5^2 + 1^2} = 2.318$. However, using the *squared* Euclidean distance (SED) assigns a higher cost to individual long travel distances and subsequently leads to solution 1 $SED[1 \rightarrow B, 2 \rightarrow A] = (0.2^2) + (1.5^2 + 1^2) = 3.29$ being more expensive than solution 2 $SED[1 \rightarrow A, 2 \rightarrow B] = (1.2^2) + (0.5^2 + 1^2) = 2.69$.

## S.2 Comparison of conflict resolution settings

In this section, we explore alternative settings for the conflict resolution method (in the following, our final method is named *Adaptive Munkres' Algorithm with Squared Euclidean Distances with* $w = 7$, or *AMASED7* for short) to justify its design, see Table S-1. All methods except *lossyBin* and *priorityShift* use the squared Euclidean distance. Each method was applied to our entire data set. We provide the cell loss with median quantile range (Q05 - Q95) and the processing time as mean and standard deviation in Table S-2 and Figure S-3.

Table S-1: Tested conflict resolution settings.

| **Conflict resolution method** | **Description** |
|---|---|
| lossyBin | No conflict resolution after initial binning, deletion of excess cells at conflicts. |
| priorityShift | Assigns the cell closest to the grid node to that grid node, moves all other conflicting cells to available nearest neighbor grid nodes in the local 3x3 neighborhood, does not move cells that are already assigned uniquely to adjacent nodes. Uses Euclidean distances. |
| hungarianShift | Moves only the conflicting cells to available grid nodes using Munkres' algorithm in local 3 × 3 neighborhood. Uses squared Euclidean distances. |
| hungarian3 | Solves the assignment problem for all cells in the (fixed) local 3 × 3 neighborhood around a conflicting grid node. Uses squared Euclidean distances. |
| hungarian5 | Solves the assignment problem for all cells in the (fixed) local 5 × 5 neighborhood around a conflicting grid node. Uses squared Euclidean distances. |
| hungarian7 | Solves the assignment problem for all cells in the (fixed) local 7 × 7 neighborhood around a conflicting grid node. Uses squared Euclidean distances. |
| AMASED5 | Munkres' algorithm with maximum adaptive window size $w_{max} = 5$. Uses squared Euclidean distances. |
| **AMASED7 (our method)** | Munkres' algorithm with maximum adaptive window size $w_{max} = 7$. Uses squared Euclidean distances. |

Table S-2: Comparison of conflict resolution settings. Color codes indicate poor (red), average (white), good (yellow) and very good (green) performance.

| **Conflict resolution method** | **Cell loss [%]** *median (Q5 - Q95)* | **processing time [s]** *mean +/- standard deviation* |
|---|---:|---:|
| lossyBin | 11.4 (8.0 - 15.3) | **0.1 +/- 0.2** |
| priorityShift | 0.0 (**0.0** - 0.5) | 0.9 +/- 0.4 |
| hungarianShift | **0.0 (0.0** - 0.1) | 0.7 +/- 0.3 |
| hungarian3 | 0.1 (**0.0** - 0.9) | 1.1 +/- 0.6 |
| hungarian5 | **0.0 (0.0** - 0.1) | 4.1 +/- 3.9 |
| hungarian7 | **0.0 (0.0 - 0.0)** | 18.1 +/- 22.6 |
| AMASED5 | **0.0 (0.0** - 0.1) | 1.6 +/- 1.5 |
| **AMASED7 (our method)** | **0.0 (0.0 - 0.0)** | 1.7 +/- 3.9 |

Figure S-3 shows the cell loss and processing time of each method. Notably, *lossyBin* is the fastest method while both *hungarian5* and *hungarian7* are slower than AMASED5 and AMASED7, as expected. AMASED7 was used as the conflict resolution for Cell2Grid in all shown experiments as it had the lowest cell loss with acceptable processing time. Figure S-4 shows that the processing time of this method increases exponentially with the number of cells in each image.

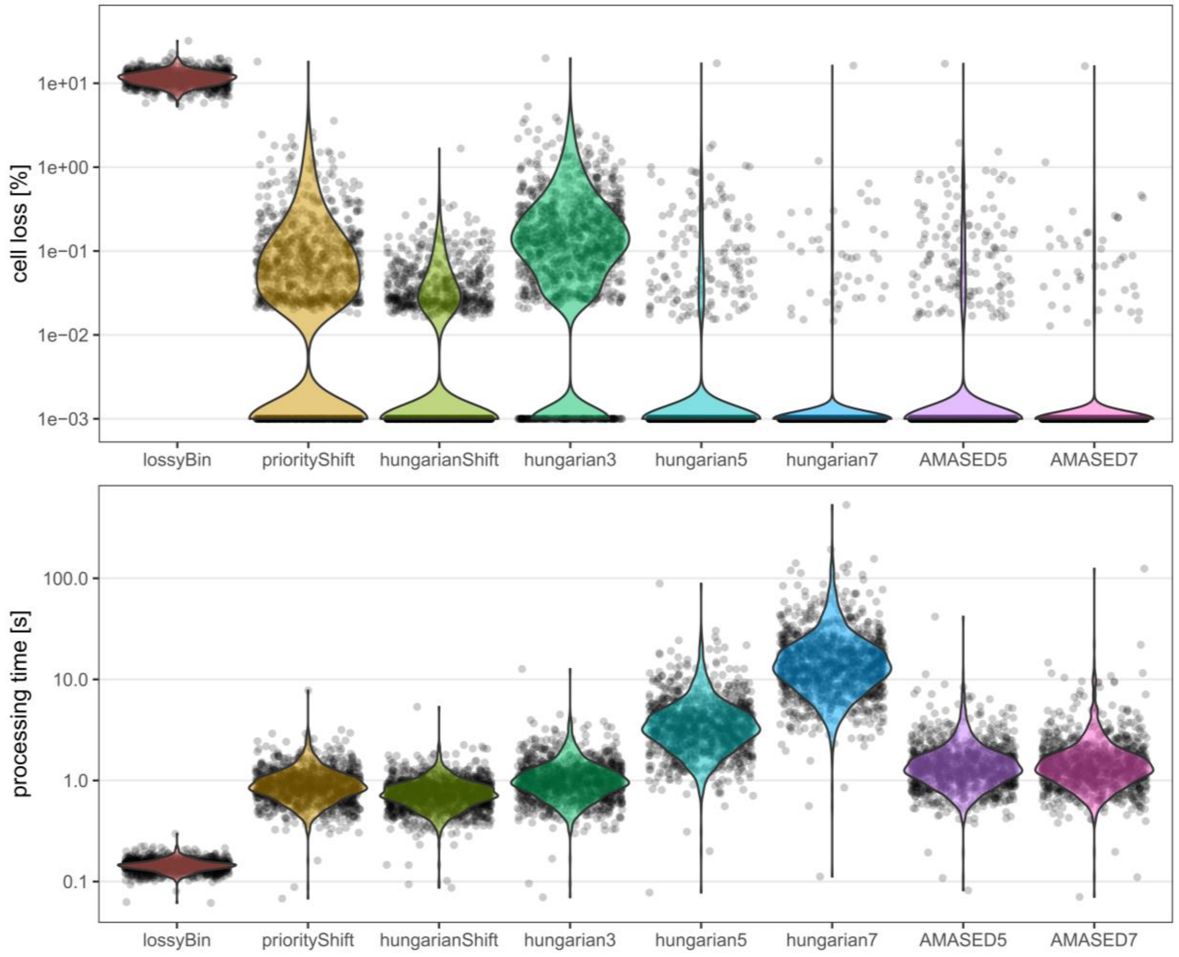


Figure S-3: Cell loss and processing time for different conflict resolution methods using a target grid of 5 $\mu m$. Zero cell loss is represented by $10^{-3}$ in log-scale. We chose AMASED7 as the default method for conflict resolution in Cell2Grid for all experiments shown in the main text.

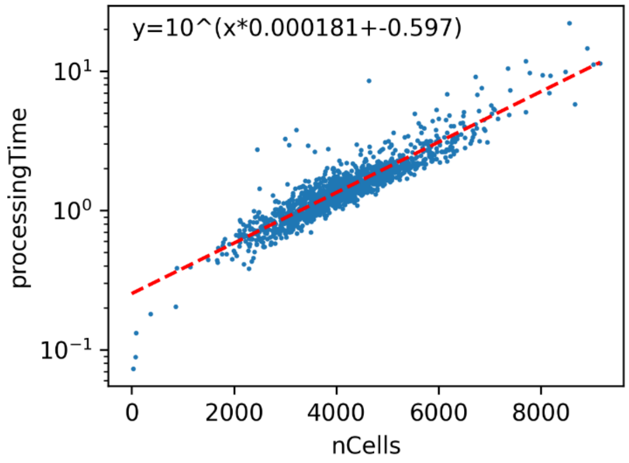


Figure S-4: Cell2Grid processing time for each individual image (blue dots) depending on the number of cells in the image using AMASED7 for conflict resolution.

## *S.3 Choosing the target grid spacing $d$*

As illustrated, the target grid spacing parameter $d$ defines the compression ratio of Cell2Grid. In our experiments, we used a target grid spacing of size $d = 5$ $\mu m$, which is comparable to the size of a lymphocyte[93], but other values are possible. Large values for $d$ (i.e., coarse grids)

provide a higher compression ratio but lead to an increased number of assignment conflicts, some of which may lead to the deletion of cells. Smaller values on the other hand create increasingly sparse Cell2Grid images and small compression ratios. Ideally, the largest value for $d$ without excessive cell loss should be used. This section provides several boundaries for an adequate choice of $d$. For illustration, this section uses empirical values from the data set introduced in the main text.

**Using a desired compression ratio**

If a specific spatial compression ratio R of a single image channel is desired, the target grid spacing is simply $d = s\sqrt{R}$, with $s$ being the original image pixel resolution. When the original image consists of $c$ color channels and $f$ cell features are extracted during Cell2Grid, this gets modified to $d = s\sqrt{R}\left(\frac{f}{c}\right)$. In our example with $f = c = 6$ and $s = 0.5\mu m$, a desired compression ratio of $R = 100$ leads to $d = 5\ \mu m$.

**Using the empirical average cell area**

Using the empirical average cell area $A_{avg}$ obtained from cell segmentation we may assume dense packing of spherical cells with a diameter $2\sqrt{A_{avg}/\pi}$. We divide by $\sqrt{2}$ to account for cells lined up unfavorably (diagonally) to the grid to obtain the target grid spacing estimate $\sqrt{2A_{avg}/\pi}$. Using our empirical data ($A_{avg} = 42\ \mu m^2$) yields an upper boundary of $d < 5.17\ \mu m$.

**Using expected inflection point of empty grid nodes and cells in conflict**

For any given cell density $\rho$, the inflection point of the expected fraction of cells in conflict $C_{conf}(d)$ (Eq. 3) can be used as an upper boundary for $d$. This point coincides with the inflection point of the expected fraction of empty grid nodes and therefore represents the point of steepest increase in conflicts and steepest decrease of empty grid nodes. At higher values for $d$ the target grid becomes densely populated and cell loss starts to increase simultaneously. For our empiric data, this upper boundary is $d < 6.3\mu m$.

**Maximum tolerable cell loss**

Using the expression for expected cell loss (Eq. 7), we can define a maximum tolerable cell loss, leading to the largest acceptable target grid spacing $d$. For our data, 1% acceptable cell loss leads to an upper boundary of $d < 6.7\ \mu m$.

### *S.4 Using additional cell features*

Figure 8 in the main text visualizes the mean marker values over entire cells as a false-color image. However, other cell features can be used in Cell2Grid output, including the marker distribution over the cell (min, max and standard deviation) as well as size and shape of the cell and its nucleus. The possible features for Cell2Grid output channels include every cell-based feature calculated during cell segmentation. As an example, Figure S-5 illustrates CD3 marker distribution features and cell shape parameters.

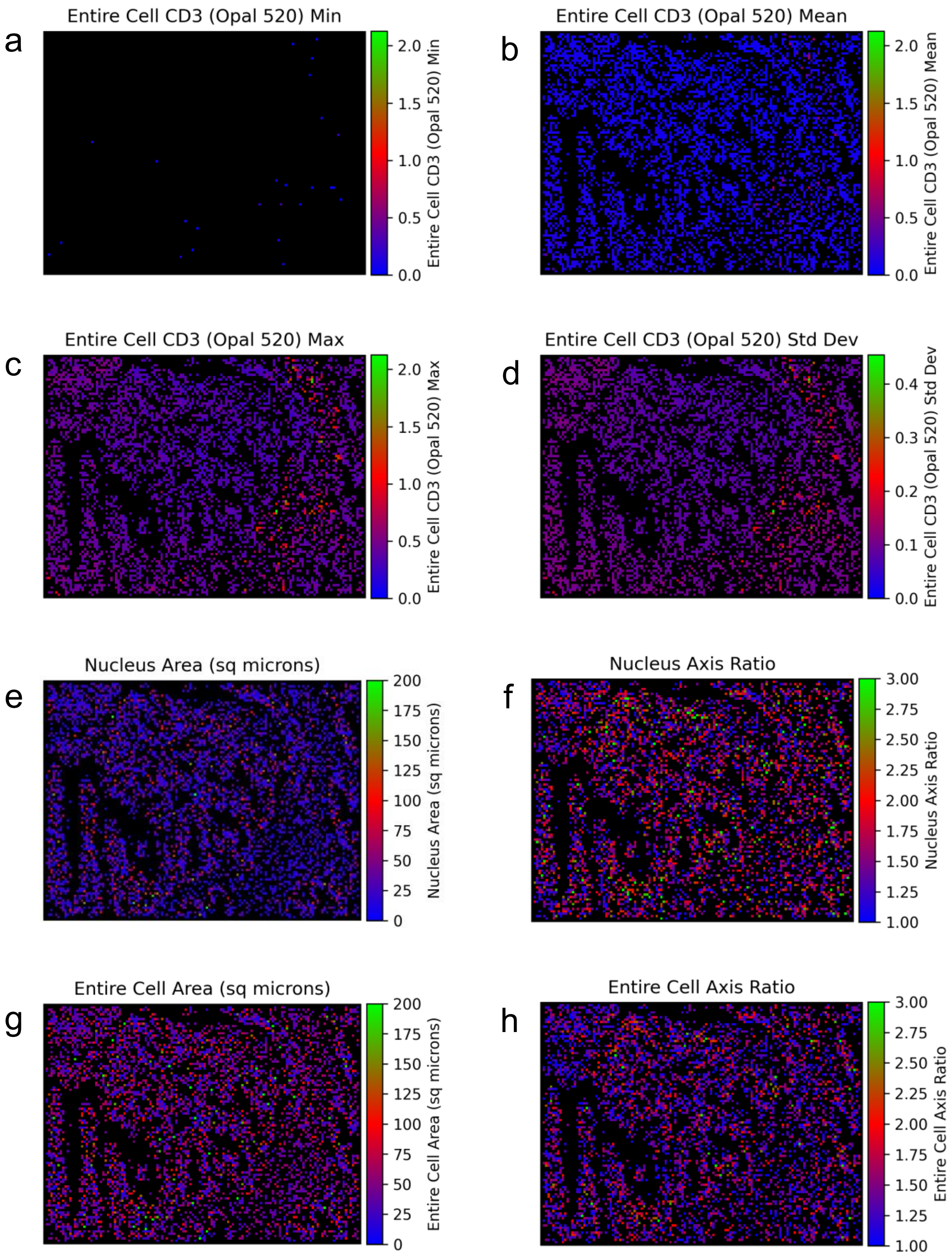


Figure S-5: Additional, non-default cell features for the same image as shown in Figure 8 of the main text. Top two rows show CD3 marker distribution features (minimum, mean, maximum, standard deviation), bottom two rows show cell shape features (area in µm² and axis ratio of nucleus and entire cell, respectively).

In addition to the results shown in the main text, we investigate below how the features of Figure S-5 change for our entire image data set after applying Cell2Grid (see Figure S-6 and Figure S-7). Since these features are only dependent on the presence of cells and not their position, they are only influenced by potential cell loss during conflict resolution.

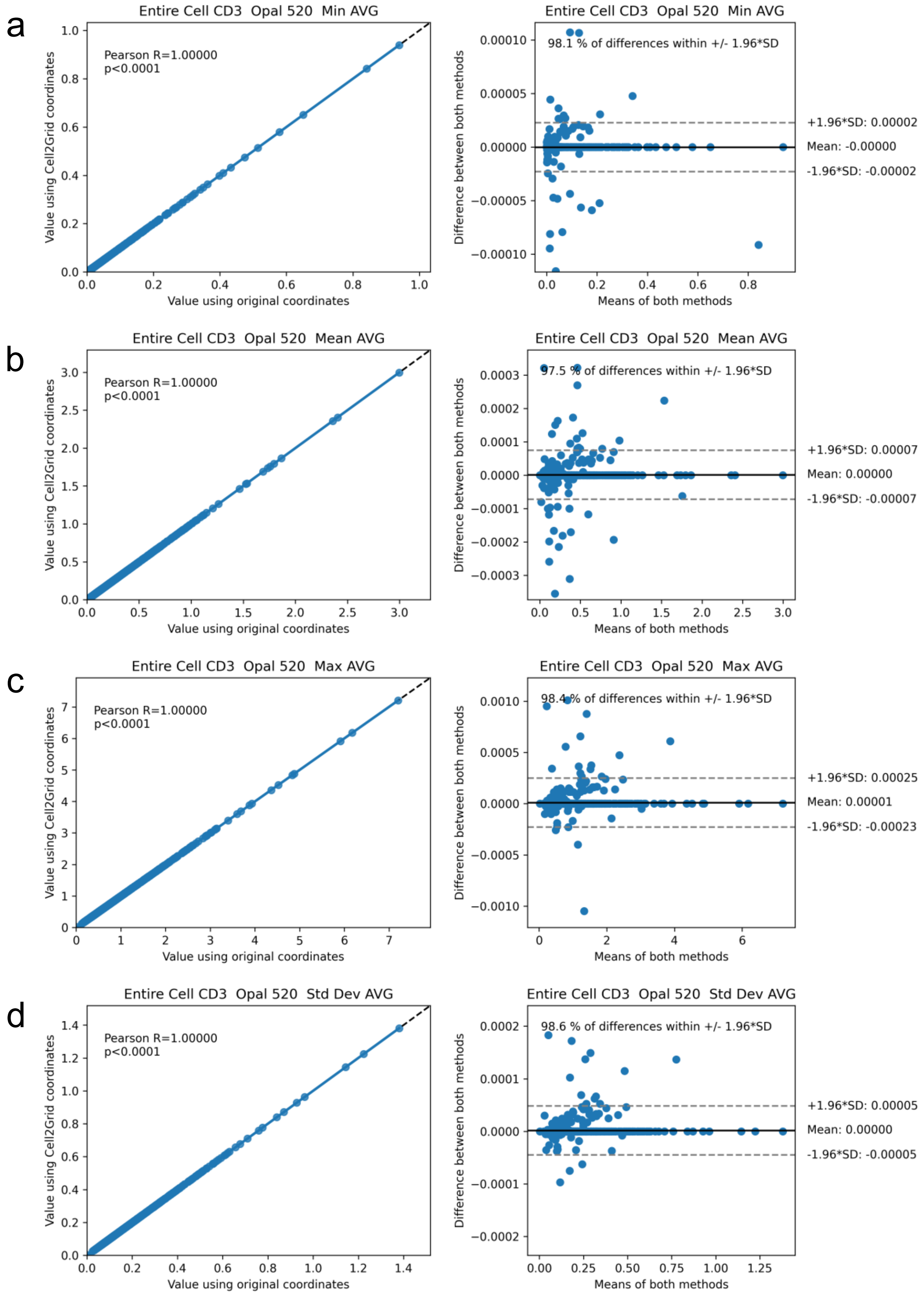


Figure S-6: Regression (left column) and Bland-Altman plots (right column) for CD3 marker distribution features of cells shown as average values per image. From top to bottom: minimum, mean, maximum, and standard deviation.

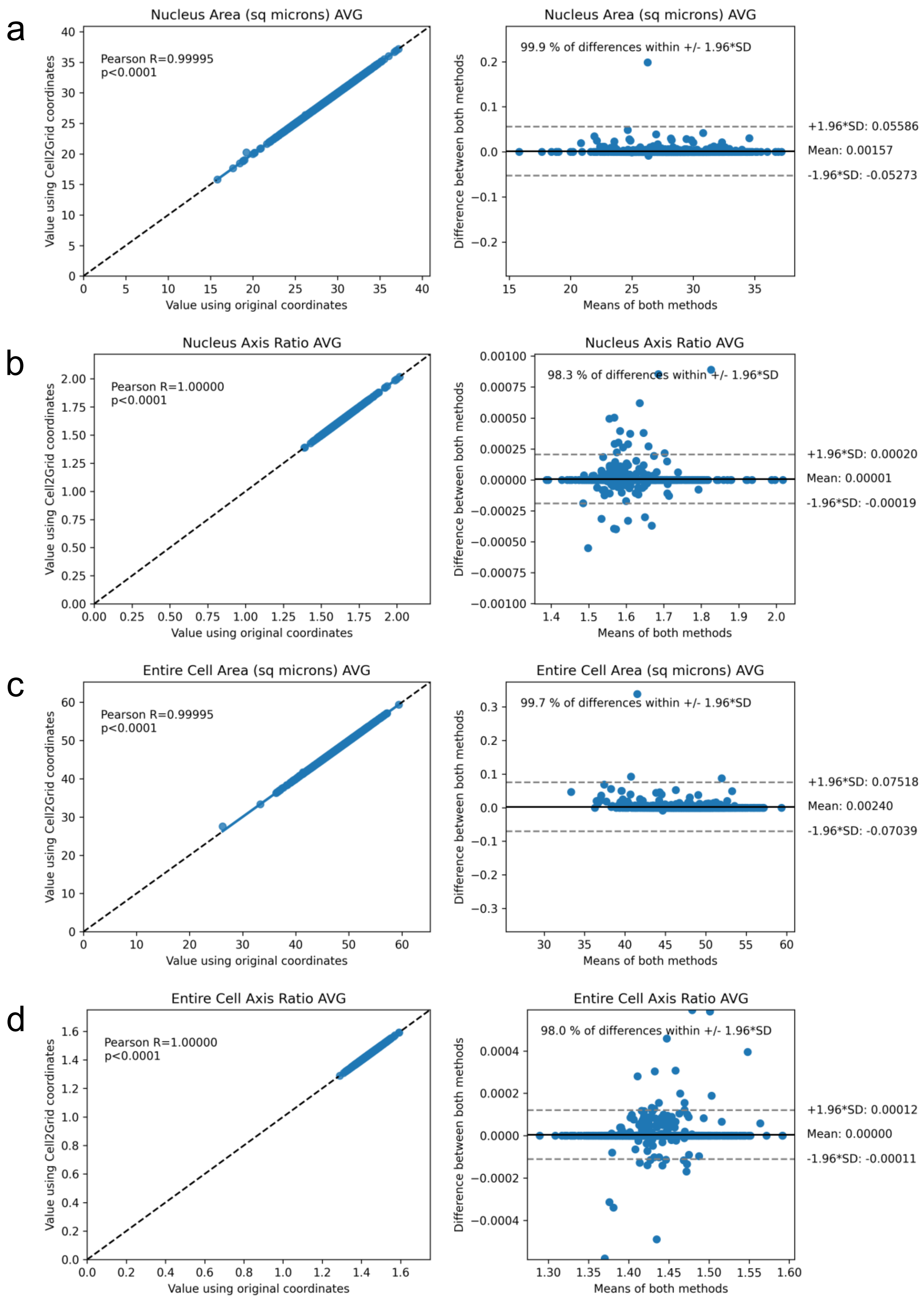


Figure S-7: Regression (left column) and Bland-Altman plots (right column) for cell shape distributions shown as average (AVG) values per image. Top to bottom: nucleus area (μm²), nucleus axis ratio, entire cell area (μm²), entire cell axis ratio.

As can be seen in Figure S-7, per-image average values for cell shape properties remain stable after applying Cell2Grid, with more than 98 % of values falling within the 1.96*Standard Deviation interval for all features. Notably, nucleus and cell size average values tend to be

shifted towards higher values in Cell2Grid, indicating that cell loss typically occurs in high-density regions that contain smaller cells on average. This tendency for losing small cells results into slightly higher average cell sizes in Cell2Grid data.

### *S.5 Data augmentation for Cell2Grid images*

As outlined in the main text, Cell2Grid images use a one-pixel-equals-one-cell concept. While it simplifies interpretation of the images, it complicates conventional data augmentation methods that involve interpolation of pixels, like rotations, shearing, zooming and other deformations. However, some of them can be replaced with discrete versions that only move pixels to new locations without altering their values by interpolation.

#### Discrete shearing

Shearing an image parallel to the x-axis using an angle $\phi$ can be expressed using a transformation matrix:

$$S = \begin{pmatrix} 1 & s \\ 0 & 1 \end{pmatrix}$$

with $s = \tan \phi$ [94]. This can be discretized by calculating the row-wise displacement $t$ of each pixel. The displacement of each row is rounded to the next integer such that each row is shifted by a pixel-discrete distance. This raster shearing[95] ensures that no pixel value needs to be interpolated but that pixels of the input image are simply moved to a different location.

#### Discrete rotation

Arbitrary rotations around an angle $\theta$ can be expressed as a chain of three independent shearing operations, known as *raster rotation*[95]:

$$R = \begin{pmatrix} \cos\theta & -\sin\theta \\ \sin\theta & \cos\theta \end{pmatrix} = \begin{pmatrix} 1 & a \\ 0 & 1 \end{pmatrix} \begin{pmatrix} 1 & 0 \\ b & 1 \end{pmatrix} \begin{pmatrix} 1 & c \\ 0 & 1 \end{pmatrix} = \begin{pmatrix} 1 + ab & a + abc + c \\ b & 1 + bc \end{pmatrix}$$

with $b = \sin\theta$ for the shearing in y-direction and $a = c = -\tan\theta/2$ for the two shearing operations in x-direction. Each individual shearing can be carried out as a discrete shearing.

### *S.6 Additional neural network experiments*

This section presents additional results for different model settings for the colon cancer relapse prediction task. We tested different learning rates for both CNN architectures and explored different weight initialization settings of the convolutional layers in VGG, see Figure S-8. Initialization of all convolutional layers except for the first one was either random or with weights pre-trained on the ImageNet data set[6,65,66]. Pre-trained layer weights were either kept fixed or were trained alongside all other network weights. Experiments with learning rate e-5 were conducted but are not shown here due to low prediction accuracy. Results are shown in Figure S-9.

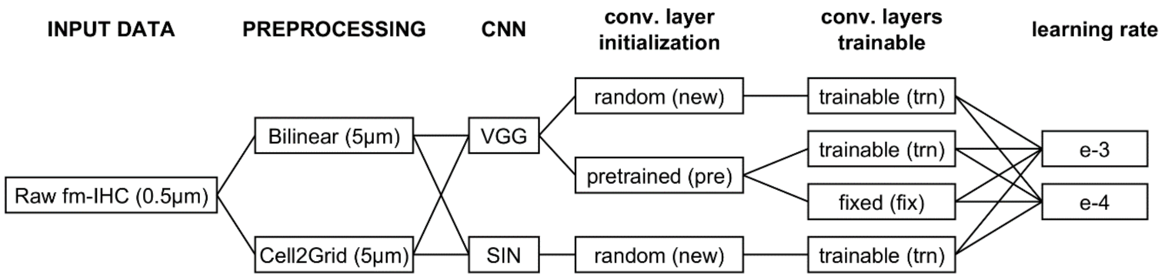


Figure S-8: Experiment setup for additional model variations.

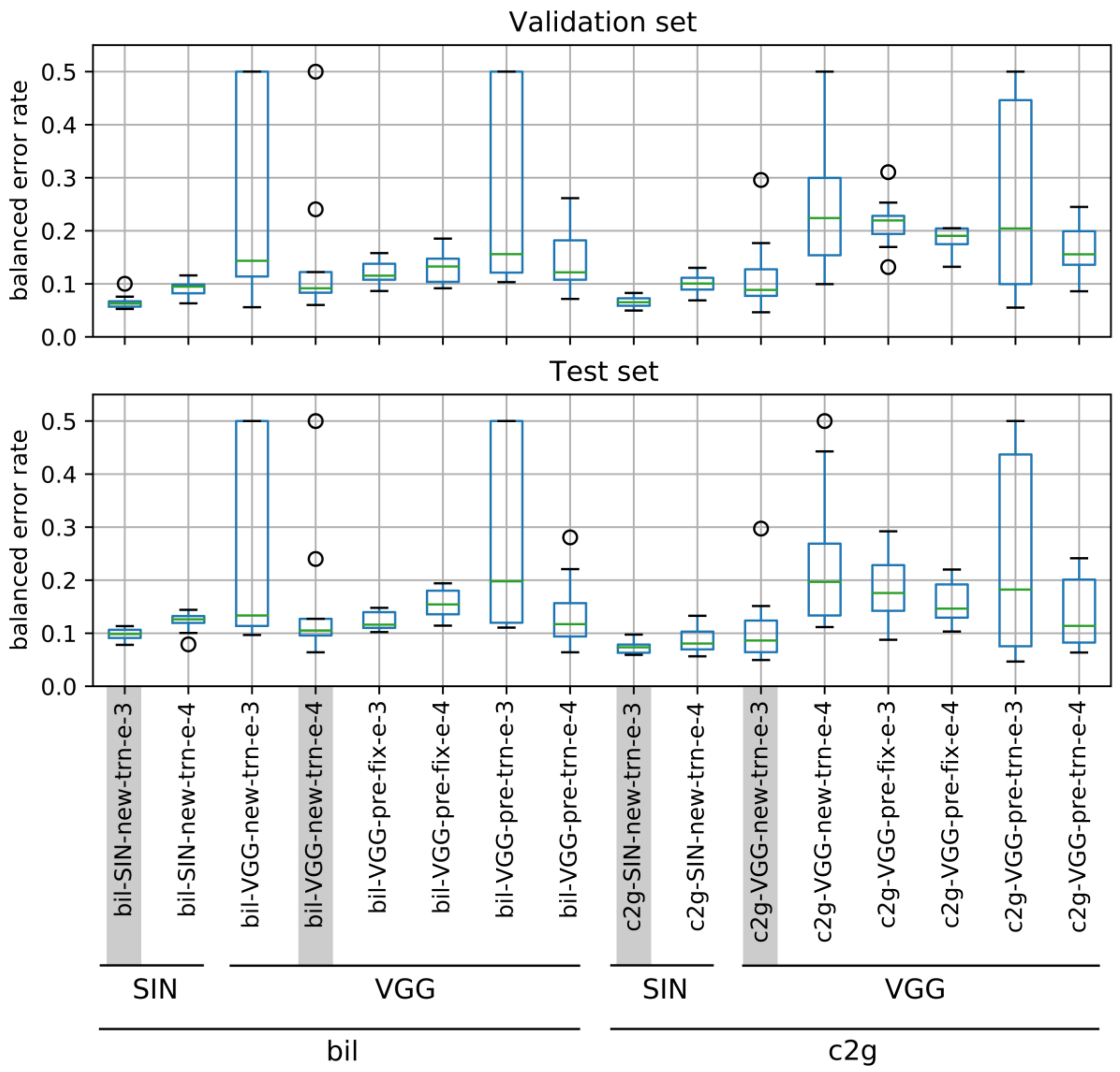


Figure S-9: Validation and test set error rates for all trained models (lower is better), 10 repeated runs shown by a single boxplot. Gray boxes indicate model settings with best validation set performance in their respective group and are sown in the main text. Abbreviations: bil (bilinear rescaling); c2g (Cell2Grid); new (network weights initialized randomly); pre (pretrained network weights); trn (pretrained weights are trainable); fix (pretrained weights kept fixed during training); e-3 and e-4 indicate learning rate.

*S.7 Literature*


6. Deng J, Dong W, Socher R, Li L, Kai Li, Li Fei-Fei. ImageNet: A large-scale hierarchical image database. In: *2009 IEEE Conference on Computer Vision and Pattern Recognition*. ; 2009:248-255.

46. Jaume G, Pati P, Foncubierta-Rodriguez A, et al. Towards Explainable Graph Representations in Digital Pathology. *arXiv [csCV]*. Published online July 1, 2020. http://arxiv.org/abs/2007.00311

55. Munkres J. Algorithms for the Assignment and Transportation Problems. *Journal of the Society for Industrial and Applied Mathematics*. 1957;5(1):32-38.

65. Chollet F, Others. Keras. Published online 2015. https://github.com/fchollet/keras

66. Kieffer B, Babaie M, Kalra S, Tizhoosh HR. Convolutional neural networks for histopathology image classification: Training vs. Using pre-trained networks. In: *2017 Seventh International Conference on Image Processing Theory, Tools and Applications (IPTA)*. ; 2017:1-6.

90. Kuhn HW. The Hungarian method for the assignment problem. *Nav Res Logist Q*. 1955;2(1-2):83-97.

91. Bourgeois F, Lassalle JC. An extension of the Munkres algorithm for the assignment problem to rectangular matrices. *Commun ACM*. 1971;14(12):802-804.

92. Tomizawa N. On some techniques useful for solution of transportation network problems. *Networks*. 1971;1(2):173-194.

93. Wood EJ. Cellular and molecular immunology (5th ed.): Abbas A. K., and Lichtman, A. H. *Biochem Mol Biol Educ*. 2004;32(1):65-66.

94. Süße H, Rodner E. *Bildverarbeitung Und Objekterkennung: Computer Vision in Industrie Und Medizin*. Springer Vieweg, Wiesbaden; 2014.

95. Paeth AW. A fast algorithm for general raster rotation. In: *Proceedings on Graphics Interface '86/Vision Interface '86*. Canadian Information Processing Society; 1986:77-81.
